# Development of a Hierarchical Support Vector Regression-Based In Silico Model for Caco-2 Permeability

**DOI:** 10.3390/pharmaceutics13020174

**Published:** 2021-01-28

**Authors:** Giang Huong Ta, Cin-Syong Jhang, Ching-Feng Weng, Max K. Leong

**Affiliations:** 1 Department of Chemistry, National Dong Hwa University, Shoufeng, Hualien 974301, Taiwan; 810812203@gms.ndhu.edu.tw (G.H.T.); 610512002@gms.ndhu.edu.tw (C.-S.J.); 2 Department of Physiology, School of Basic Medical Science, Xiamen Medical College, Xiamen 361023, China; cfweng@gms.ndhu.edu.tw

**Keywords:** intestinal absorption, intestinal permeability, human colon carcinoma cell layer (Caco-2), hierarchical support vector regression (HSVR)

## Abstract

Drug absorption is one of the critical factors that should be taken into account in the process of drug discovery and development. The human colon carcinoma cell layer (Caco-2) model has been frequently used as a surrogate to preliminarily investigate the intestinal absorption. In this study, a quantitative structure–activity relationship (QSAR) model was generated using the innovative machine learning-based hierarchical support vector regression (HSVR) scheme to depict the exceedingly confounding passive diffusion and transporter-mediated active transport. The HSVR model displayed good agreement with the experimental values of the training samples, test samples, and outlier samples. The predictivity of HSVR was further validated by a mock test and verified by various stringent statistical criteria. Consequently, this HSVR model can be employed to forecast the Caco-2 permeability to assist drug discovery and development.

## 1. Introduction

Clinically, the majority of drugs are orally administered [1]. Prior to reaching the blood circulation system, the administered pharmaceutical agents have to pass through the intestinal barrier via passive diffusion, active uptake, and/or efflux transport processes [2,3,4], as illustrated by Figure 10.2 of Proctor et al. [2]. In passive diffusion, drug molecules can permeate the epithelial cell layers through the transcellular pathway, in which they penetrate through the cell membrane, or the paracellular pathway, in which they can cross the epithelial cell layer through the tight junction between cells [5]. The significance of active transporters on intestinal absorption has been detailed elsewhere [6]. Principally, active transport can be modulated by the efflux transporters of the ATP-binding cassette (ABC) family as well the influx transporters of the solute carrier (SLC) family [6], of which the efflux transporters can pump the administrated drugs out of enterocytes, leading to the reduction of the accumulated concentration, whereas the influx can enhance the intestinal uptake, resulting in the increased drug accumulation [7]. Of various active influx and efflux transporters, P-glycoprotein (P-gp), also termed multidrug resistance 1 protein (MDR1/encoded by *ABCB1* gene), breast cancer resistance protein (BCRP/*ABCG2*), organic anion transporting polypeptide 2B1 (OATP2B1/*SLCO2B1*), and peptide transporter 1 (PEPT1/*SLC15A1*) play predominant roles in intestinal absorption [8].

Passive diffusion depends on a number of physicochemical properties, whereas active transport relies on the characteristics of specific binding sites on the transport proteins [9]. The uncharged and modest hydrophobic drugs such as testosterone [10] can permeate through the membrane. Conversely, it is very difficult for highly hydrophobic molecules to get across cells, since they can be adhered to the membrane [5]. On the other hand, hydrophilic drugs such as mannitol predominantly pass through the paracellular pathway [10].

Of various drug absorption, distribution, metabolism, elimination, and toxicity (ADME/Tox) properties, drug absorption plays a pivotal role in drug discovery, since they substantially contribute to the earlier preclinical go/no-go decisions for the drug candidates [10,11] to achieve the “fail fast, fail early” paradigm [12]. As such, numerous in vivo and in situ assays have been developed to evaluate the intestinal absorption [13,14]. For instance, the in situ single-pass intestinal perfusion (SPIP) model measures the appearance of the drug in plasma after intravenous and intraintestinal drug administration [13,15]. The drug is orally administrated or directly given into the intestine or stomach in some animal species in in vivo assay [13,14,16].

In addition to in vivo and in situ assays, various in vitro assays have been devised, since they have more advantages such as low cost and time efficiency as compared with their in situ and in vivo counterparts [15]. Of various in vitro assays to evaluate intestinal absorption, human colon carcinoma monolayer cells (Caco-2) [3], parallel artificial membrane permeability (PAMPA) [17,18], and Madin–Darby canine kidney cells (MDCK) [19] are most frequently used. In fact, a comprehensive drug absorption profile should include the Caco-2, MDCK, and PAMPA permeability data to explore drug solubility and bioavailability [20]. Moreover, Caco-2, which can be adopted to evaluate the drug permeability through the cytoplasm (transcellular uptake) or between cells (paracellular uptake) and active transport [6], has become the golden standard for predicting intestinal drug permeability and absorption because of its similarity in morphology and function with human enterocytes [21,22,23]. The Caco-2 protocol has been clearly described in detail by Hubatsch et al. As compared with the biological membrane, the Caco-2 system still suffers from a range of disadvantages such as high technical complexity, the limitations related to the differences between cell monolayers and intestinal membrane structurally and functionally [24], in addition to its long culture periods (21‒24 days) with the significantly extensive costs, contributing to the major concerns in practical applications [21,25].

The Caco-2 permeability is normally expressed by the apparent permeability coefficient (*P*_app_), in which the drug solution is added to the apical side, viz. the donor compartment, and the *P*_app_ value in the basolateral side, viz. the receiver compartment, is measured [23]

(1) $$P_{\mathrm{app}}=\frac{dQ}{dt}\times \frac{1}{\left(A\times C_{0}\right)}$$

where *dQ*/*dt* is the linear appearance rate of mass in the receiver solution transported during sink conditions, *A* is the membrane surface area, and *C*_0_ is the initial concentration at the donor compartment [26]. However, it is not uncommon to observe in vitro permeability variations among different from research groups, because the cultured cells can vary based on culture conditions, passage number, monolayer age, seeding density, and stage of differentiation [27,28], as exemplified by those compounds listed in Table 3 of Lee et al. [29]. Furthermore, Yamashita et al. have found that the different pH values of apical medium and the different solvents can produce different drug absorption values [30]. For instance, the *P*_app_ values of alprenolol are (6.06 ± 0.18) × 10^‒6^ cm/s and (30.0 ± 1.8) × 10^‒6^ cm/s at pH 6.0 and pH 7.4, respectively. More examples of *P*_app_ variations at different pH values can be found in Table 1 of Yamashita et al. [30].

In silico technologies have become an essential component in drug discovery and development according to the fact that they can provide guidance in the early stages in the drug discovery process such as the activity classification (high/moderate/poor) or quantitative predictions [31,32]. As such, a great number of in silico models have been established to predict the ADME/Tox properties [33]. The relationship between biological activity and chemical characteristics can be established by quantitative structure–activity or structure–property relationships (QSAR and QSPR) [34]. Numerous QSAR models have been generated to predict Caco-2 permeability based on a variety of physicochemical and physiological descriptors [35,36,37,38,39,40,41,42,43,44,45,46,47,48,49,50,51]. Nevertheless, the difficulties in developing sound in silico models to predict the intestinal permeability still remain unanswered mainly due to the fact that Caco-2 permeability is a dramatically perplexing process that can take place through numerous non-linear routes (vide supra).

More specifically, the ABC transporters, which are efflux transporters, can reduce the drug absorption, whereas the SLC transporters, which are influx transporters, can enhance the drug uptake, leading to the decrease and/or increase of drug absorption, respectively. In fact, such controversy can establish a paramount barrier in model development. For instance, the number of aromatic rings (*n*_Ar_) can enhance the compound hydrophobicity [52] and facilitate the passive diffusion consequently. Conversely, *n*_Ar_ is also an important feature for P-gp substrate recognition and modulates the compound efflux correspondingly [53]. Thus, *n*_Ar_ can simultaneously affect the active efflux and passive diffusion.

It is exceedingly difficult, if not nearly impossible, to derive a robust in silico model, which can properly render the complex relationships between the selected descriptors and Caco-2 permeability. However, the hierarchical support vector regression (HSVR) scheme, which is an innovative machine learning-based scheme initially developed by Leong et al. [54], can properly address the complicated and varied dependencies of descriptors that, in turn, can be greatly contributed to its advantageous features of both a local model and a global model, namely wider coverage of applicability domain (AD) and a higher capability of prediction, respectively. When comparing with most theoretical models, which are vulnerable to the outliers that represent mathematic extrapolations, HSVR can still show consistent performance, as demonstrated elsewhere [1,54,55,56,57]. Herein, the objective of this study was to develop an in silico model based on the HSVR scheme to predict Caco-2 permeability in conjunction with previously published PAMPA permeability, intestinal absorption, and MDCK efflux in silico models [1,55,57] to facilitate drug discovery and development, since medicinal chemists can employ these models to predict the drug absorption of (virtual) hit compounds as well as drug metabolism and pharmacokinetics (DM/PK) scientists can adopt these models to prioritize the lead compounds.

## 2. Materials and Methods

### 2.1. Data Collection

The *P*_app_ values were collected from the various sources after a comprehensive literature search [22,23,58,59,60,61,62,63,64,65,66]. Assay systems were carefully scrutinized to ensure data consistency, since various assay conditions such as pH value and solvent system, for example, can affect the Caco-2 permeability [30]. Only *P*_app_ values, which were measured in the Hank’s balanced salt solution (HBSS) buffer and 4-(2-hydroxyethyl)-1-piperazineethanesulfonic acid (HEPES) including ca. 1% dimethylsulfoxide (DMSO) at pH 7.4 were chosen in this study. The average *P*_app_ value was selected to warrant better consistency in case there was more than one *P*_app_ value for a given compound within a near range. Finally, 144 compounds were chosen in this study and their corresponding logarithm *P*_app_ values, simplified molecular input line entry system (SMILES) strings, Chemical Abstracts Service (CAS) registry numbers, and references to the literature are listed in Table S1.

### 2.2. Molecular Descriptors

The density functional theory (DFT), Becke 3-parameter Lee–Yang–Parr (B3LYP) method was employed to do full geometry optimization by the Gaussian package (Gaussian, Wallingford, CT, USA) for all recruited samples with the selection of basis set 6-31G (*d,p*). The solvent system was taken into consideration by the polarizable continuum model (PCM) [67,68]. The atomic charges, upon which the dipole moments depend, were calculated by the molecular electrostatic potential (MEP) [69]. The frontier orbitals energies, namely the highest occupied molecular orbital energy (*E*_HOMO_) and the lowest unoccupied molecular orbital energy (*E*_LUMO_), molecular dipole (*µ*), as well as the maximum absolute component of *µ* (|*µ*|_max_) were also recovered from the optimization calculations.

In total, more than 100 descriptors, which feature one-, two-, and three-dimensional ones and can be categorized into a variety of classes consisting of topological descriptors, electronic descriptors, thermodynamic descriptors, structure descriptors, spatial descriptors, and *E*-state indices, were enumerated by Discovery Studio (BIOVIA, San Diego, CA, USA) and E-Dragon (available at the website http://www.vcclab.org/lab/edragon/). The logarithm of the *n*-octanol–water partition coefficient at pH 7.4 (log *P*) was calculated by *XLOGP3* of SwissADME (available at the website http://www.swissadme.ch/index.php). Furthermore, the cross-sectional area (CSA), which has been implicated in membrane permeability [70,71], was calculated using the method modified by Muehlbacher et al. [72]. The collected compounds were divided into 4 ion classes [73], namely zwitterion, base, acid, and neutral ions according to their p*K*_a_ values. The neutral ions only have one p*K*_a_ value, the zwitterion ions are those whose strongest acidic p*K*_a_ values are larger than 7 and the strongest basic ones are smaller than 7, the acidic ions have all their p*K*_a_ values smaller than 7, whereas the basic ions have all their p*K*_a_ values larger than 7.

### 2.3. Descriptor Selection

Descriptor selection was initially executed by removing those descriptors missing more than one molecule or displaying little or no distinction among all molecules. Furthermore, the Spearman’s matrix between calculated descriptors was constructed to minimize the chance of spurious correlations, and those descriptors with intercorrelation values of *r*^2^ > 0.80 were discarded, since the threshold was proposed by Topliss and Edwards [74]. In this study, a more conservative value of *r*^2^ ≥ 0.64 was taken to further ensure the quality of derived models.

Descriptor values can span a wide range due to their diverse nature (vide supra). It is of necessity to transfer descriptors into a more consistent range to decrease the chance of descriptors with broader ranges overriding those with narrower ranges [75]. Accordingly, descriptors were subjected to normalization by centering and scaling

(2) $${\hat{x}}_{ij}^{}=\frac{x_{ij}-\langle x_{j}\rangle }{\sqrt{\sum_{i=1}^{n}(x_{ij}-{\langle x_{j}\rangle )}^{2}/(n-1)}}$$

where $x_{ij}$ and ${\hat{x}}_{ij}$ symbolize the *j*th original and normalized descriptors of the *i*th molecule, respectively; $\langle x_{j}\rangle$ is the average value of the original *j*th descriptor; and *n* is the number of molecules.

The descriptor selection is of pivotal importance in the performance of QSAR models [76]. Thus, genetic function approximation (GFA) bundled in the QSAR module of Discovery Studio was used for the initial descriptor because of its effectiveness and efficiency [77]. The recursive feature elimination (RFE) scheme was adopted for additional selection, in which the model was repeatedly generated by all but one descriptor. The descriptor, which had the less contribution in predictive performance, was removed after ranking their contributions [78].

### 2.4. Dataset Selection

It is not uncommon to identify the outliers and remove them from data collection for model development [79]. As such, outliers were recognized by inspecting molecular distribution in the chemical space [80], which was created by principal components (PCs) using the Diverse Molecules/Principal Component Analysis embedded in Discovery Studio, followed by discovering the outliers.

The remaining molecules were arbitrarily allocated into the training set and test set with an about 4:1 portion as recommended [81] to generate and verify the built model, respectively, using the Diverse Molecules/Library Analysis function within Discovery Studio. Golbraikh et al. have postulated that a sound model can be resulted only when both samples in the training set and test set can show high levels of chemical and biological similarity [82]. Thus, the data distributions in the training and test set were carefully checked to ensure the high similarity degrees biologically and chemically in both datasets.

### 2.5. Hierarchical Support Vector Regression

Leong et al. originally invented HSVR [54] which was evolved from support vector machine (SVM) proposed by Vapnik et al. [83]. Initially, SVM was designed for classification only and the regression function, termed as support vector regression (SVR), was introduced later [84]. HSVR has a higher level of predictivity and broader applicability domain (AD) as compared with SVR, since it can seamlessly combine the advantages of the local model and global model [56]. More significantly, the superiority of HSVR has been revealed by some studies [1,54,55,56,57].

The theory and fulfillment of HSVR have been delineated in detail elsewhere, and the schematic presentation of HSVR can be depicted by Figure 1 of Leong et al. [54]. Basically, an SVR ensemble (SVRE) is used to build an HSVR model, and SVR models in the ensemble are generated from different descriptor combinations and function as local models with their own ADs. Briefly, the svm-train module in *LIBSVM* (software available at http://www.csie.ntu.edu.tw/~cjlin/libsvm/) was employed to build various SVR models using those samples in the training set with different descriptor combinations and SVR run conditions. The module svm-predict in *LIBSVM* was adopted to validate the produced SVR models using the samples in the test set. Radial basis function (RBF) was the designated kernel function due to its simplicity and better functionality [85]. Both *ε*-SVR and *ν*-SVR regression functions were tested. The SVR runtime conditions including *ε*-SVR and *ν*-SVR, their associated *ε* and *ν*, the kernel width *γ*, and cost *C* were tuned by the grid-search technique.

According to the principle of Occam’s razor, i.e., the principle of parsimony, the number of descriptors selected to build SVR models should be minimized as much as possible. This principle was also applied to the construction of SVRE, which demanded the minimum number of ensemble members [86]. Initially, the combinations of two SVR models were adopted to generate the HSVR model; this process was repeated until the production of a predictive HSVR. Otherwise, the combinations of three- or even four-member SVRE were used to develop the HSVR models if the two-SVR ensembles failed to perform well.

### 2.6. Predictive Evaluation

The residual yielded by the difference between the observed value $\left(y_{i}\right)$ and the predicted value $\left({\hat{y}}_{i}\right)$ for the *i*th molecule was computed based on the following equation:

(3) $$\Delta_{i}=y_{i}-{\hat{y}}_{i}$$

In addition, standard deviation (*s*), maximum residual (∆_Max_), root mean square error (RMSE), and mean absolute error (MAE) in a dataset with *n* samples were evaluated.

(4) $$\mathrm{RMSE}=\sqrt{\sum_{i=1}^{n}{△}_{i}^{2}/n}$$

(5) $$\mathrm{MAE}=\frac{1}{n}\sum_{i=1}^{n}|\Delta_{i}|$$

Various statistic metrics were adopted to evaluate the produced models. The squared correlation coefficients including *r*^2^ and *q*^2^ in the training set and external set, respectively, were computed by the following equation.

(6) $$r^{2},\;q^{2}=1-\sum_{i=1}^{n}({\hat{y}}_{i}-y_{i}{)}^{2}/\sum_{i=1}^{n}(y_{i}-\langle \hat{y}\rangle {)}^{2}$$

where $\langle {\hat{y}}_{i}\rangle$ represents the average predicted value, and *n* is the number of samples in the dataset. The derived models were subjected to the 10-fold cross-validation using the function embedded in *LIBSVM* to give rise to the squared correlation coefficient of 10-fold cross-validation $q_{\mathrm{CV}}^{2}$. Another internal validation was carried out by the *Y*-scrambling test [87], in which the log *P*_app_ values were randomly permuted and then reapplied to the previous developed model without altering the descriptors. This process was repeated 25 times as suggested [87] to generate the average squared correlation coefficient $\langle r_{s}^{2}\rangle$.

The external dataset was evaluated predictivity by the squared correlation coefficients $q_{F1}^{2}$, $q_{F2}^{2}$, and $q_{F3}^{2}$, and the concordance correlation coefficient (*CCC*) [88,89,90,91,92,93] using *QSARINS* [94,95].

(7) $$q_{F1}^{2}=1-\sum_{i=1}^{n_{\mathrm{EXT}}}{\left(y_{i}-{\hat{y}}_{i}\right)}^{2}/\sum_{i=1}^{n_{\mathrm{EXT}}}{\left(y_{i}-\langle y_{\mathrm{TR}}\rangle \right)}^{2}$$

(8) $$q_{F2}^{2}=1-\sum_{i=1}^{n_{\mathrm{EXT}}}{\left(y_{i}-{\hat{y}}_{i}\right)}^{2}/\sum_{i=1}^{n_{\mathrm{EXT}}}{\left(y_{i}-\langle y_{\mathrm{EXT}}\rangle \right)}^{2}$$

(9) $$q_{F3}^{2}=1-\left[\sum_{i=1}^{n_{\mathrm{EXT}}}{\left(y_{i}-{\hat{y}}_{i}\right)}^{2}/n_{\mathrm{EXT}}\right]/\left[\sum_{i=1}^{n_{\mathrm{EXT}}}{\left(y_{i}-\langle y_{\mathrm{TR}}\rangle \right)}^{2}/n_{\mathrm{TR}}\right]$$

(10) $$CCC=\frac{2\sum_{i=1}^{n_{\mathrm{EXT}}}\left(y_{i}-\langle y_{\mathrm{EXT}}\rangle \right)\left({\hat{y}}_{i}-\langle {\hat{y}}_{\mathrm{EXT}}\rangle \right)}{\sum_{i=1}^{n_{\mathrm{EXT}}}{\left(y_{i}-\langle y_{\mathrm{EXT}}\rangle \right)}^{2}+\sum_{i=1}^{n_{\mathrm{EXT}}}{\left({\hat{y}}_{i}-\langle {\hat{y}}_{\mathrm{EXT}}\rangle \right)}^{2}+n_{\mathrm{EXT}}{\left(\langle y_{\mathrm{EXT}}\rangle -\langle {\hat{y}}_{\mathrm{EXT}}\rangle \right)}^{2}}$$

where $\langle y_{\mathrm{TR}}\rangle$ is the averaged observed values in the training set, $\langle y_{\mathrm{EXT}}\rangle$ and $\langle {\hat{y}}_{\mathrm{EXT}}\rangle$ are the averaged observed and predicted values in the external set, respectively; $n_{\mathrm{TR}}$ and $n_{\mathrm{EXT}}$ stand for the numbers of samples in the training set and external set, respectively.

In addition, some modified squared correlation coefficients *r*^2^ were estimated [96,97]

(11) $$r_{m}^{2}=r^{2}\left(1-\sqrt{\left|r^{2}-r_{o}^{2}\right|}\right)$$

(12) $${r^{\prime }}_{m}^{2}=r^{2}\left(1-\sqrt{\left|r^{2}-{r^{\prime }}_{o}^{2}\right|}\right)$$

(13) $$\langle r_{m}^{2}\rangle =\left(r_{m}^{2}+{r^{\prime }}_{m}^{2}\right)/2$$

(14) $$\Delta r_{m}^{2}=\left|r_{m}^{2}-{r^{\prime }}_{m}^{2}\right|$$

(15) $$\left(r^{2}-r_{o}^{2}\right)/r^{2}<0.10\;\mathrm{and}\;0.85\leq k\leq 1.15.$$

To externally evaluate the predictivity of the generated models, the most stringent criteria validation values jointly proposed by Golbraikh et al. [82], Ojha et al. [96], Roy et al. [98], and Chirico and Gramatica [89] were adopted

(16) $$r^{2},\;q_{\mathrm{CV}}^{2},\;q^{2},\;q_{\mathrm{Fn}}^{2}\geq 0.70$$

(17) $$\left|r^{2}-q_{\mathrm{CV}}^{2}\right|<0.10$$

(18) $$\left|r_{0}^{2}-{r^{\prime }}_{0}^{2}\right|<0.30$$

(19) $$r_{m}^{2}\geq 0.65$$

(20) $$\langle r_{m}^{2}\rangle \geq 0.65\;\mathrm{and}\;\Delta r_{m}^{2}<0.20$$

(21) CCC≥0.85

where *r*^2^ in Equations (15) and (18)‒(20) symbolize *r*^2^ and *q*^2^ in the training set and external set, respectively. The $q_{\mathrm{Fn}}^{2}$ in Equation (16) stands for $q_{F1}^{2}$, $q_{F2}^{2}$, and $q_{F3}^{2}$.

## 3. Results

### 3.1. Dataset Selection

**Figure 1:**
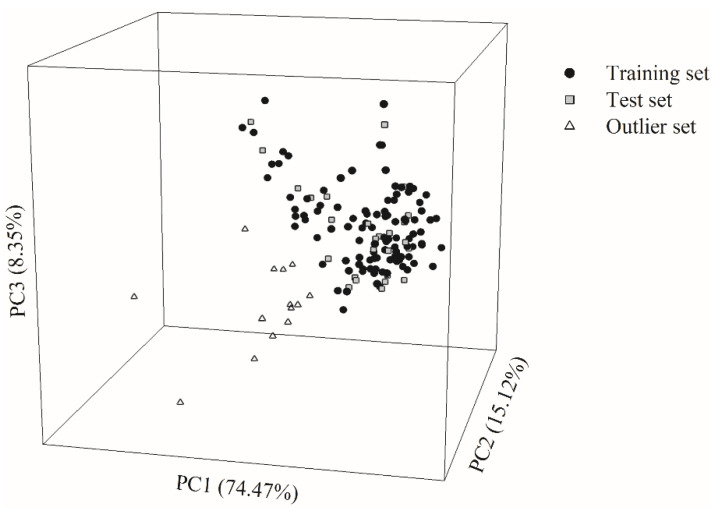
The chemical space spanned by three principle components (PCs) displays the distribution of the data samples in the training set (solid circle), test set (gray square), and outlier set (open triangle).

Of all the molecules enrolled in this study, 104 and 26 molecules were randomly selected as the training set and test set, respectively, giving rise to a ca. 4:1 ratio as suggested [81]. The chemical space with the projection of all molecules is displayed in Figure 1. Three principle components (PCs), which accounted for 97.94% of the variance in the original data, were used to create the chemical space. This figure shows that samples in the training set and test set had similar distribution in the chemical space. The high levels of the biological and chemical similarity between both datasets can be illustrated by the histograms of log *P*_app_, molecular weight (MW), surface area (SA), polar surface area (PSA), number of hydrogen bond acceptor (HBA), number of hydrogen bond donor (HBD), and *n*-octanol‒water partition coefficient (log *P*) in the density form (Figure S1). Thus, it is plausible to assert that the substantial bias did not appear in the data partition.

It is of great significance to characterize the AD of the predictive model and to exclude the outliers from data collection [94]. Various methods to detect outliers have been proposed [99]. The scheme based on the chemical similarity/dissimilarity using principle component analysis (PCA) was adopted in this study [94]. Accordingly, 14 molecules were specified as outliers, which are substantially dissimilar to those ones in both the training and test sets, as shown in the chemical space (Figure 1), from which it can be observed that they are located far from the others. The distinction between the outliers and the others can be actually recognized by the fact that they contain more than nine rings or more than 12 HBAs as compared with the other molecules.

### 3.2. SVR Models

Numerous SVR models were generated using different descriptor combinations and runtime conditions. Three SVR models, coined as SVR A, SVR B, and SVR C, were assembled to establish the SVR ensemble, which was successively utilized to generate the HSVR model by another SVR. The optimal runtime conditions of SVR A, SVR B, SVR C, and HSVR are listed in Table S2.

SVR A, SVR B, and SVR C adopted five, five, and seven descriptors, respectively, with different combinations (Table 1). These SVR models in the ensemble were assembled according to their performances on the molecules and statistical assessments in the training set and test set. Their runtime conditions and their predicted log *P*_app_ values are listed in Tables S1 and S2, respectively. Table 2 and Table 3 record their associated statistical evaluations in the training set and test set, respectively.

**Figure 2:**
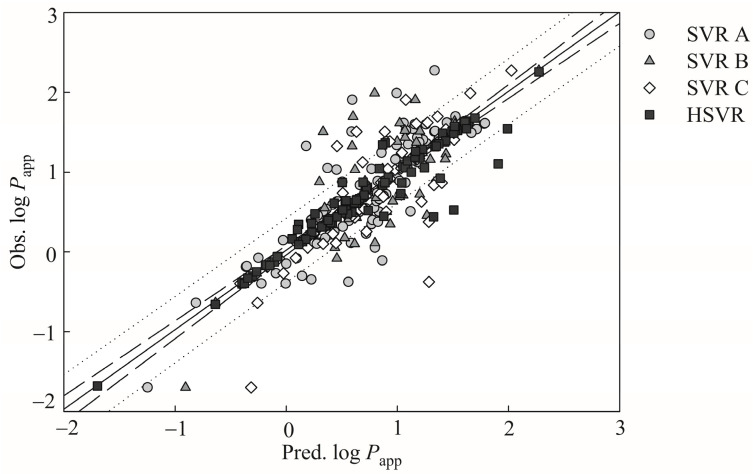
Observed log *P*_app_ versus the log *P*_app_ predicted by SVR A (gray circle), SVR B (gray triangle), SVR C (open diamond), and HSVR (solid square) for the training samples. The solid, dashed, and dotted lines represent to the HSVR regression of the data, 95% confidence intervals for the HSVR regression, and 95% confidence intervals for the prediction, respectively.

The observed versus the predicted log *P*_app_ values by SVR A, SVR B, SVR C, and HSVR are displayed by the scatter plot in Figure 2, from which it can be observed that SVR A, SVR B, and SVR C predicted the observed values well for the majority of the molecules in the training set, producing small MAE and *s* values consequently (Table 2). Moreover, it can be found from Figure 2 that the points predicted by SVR B are generally closer to the regression line than SVR A and SVR C. SVR B, consequently, gave rise to the lowest Δ_Max_ (1.19), MAE (0.17), and RMSE (0.32), and the largest *r*^2^ (0.77), suggesting that SVR B performed marginally better than SVR A and SVR C in the training set.

Furthermore, the difference between *r*^2^ and $q_{\mathrm{CV}}^{2}$ evaluated by SVR B was 0.58 when subjected to the leave-one-out cross-validation, indicating that SVR B was over-trained which, in turn, can severely limit its application. Over-training was also associated with SVR A and SVR C as manifested by their extremely low $q_{\mathrm{CV}}^{2}$ values. The $\langle r_{s}^{2}\rangle$ values produced by SVR A, SVR B, and SVR C were 0.05, 0.03, and 0.03 (Table 2), respectively, when subjected in *Y*-scrambling. These near zero values suggest that there is an almost zero chance correlation associated with those SVR models [87].

**Figure 3:**
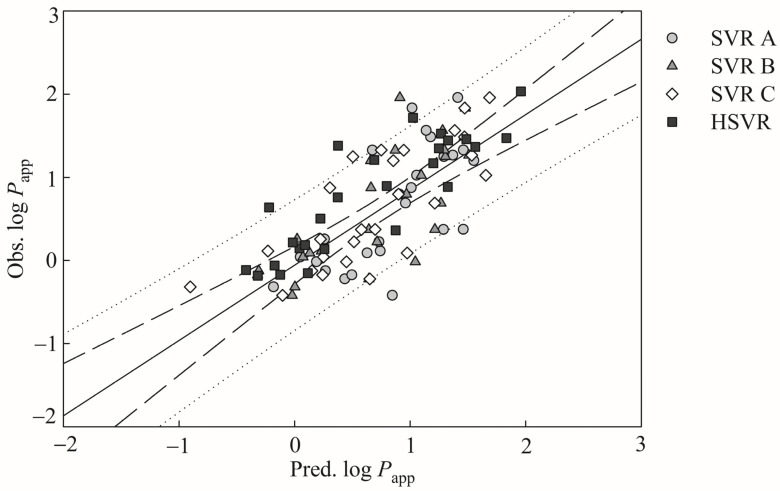
Observed log *P*_app_ versus the log *P*_app_ predicted by SVR A (gray circle), SVR B (gray triangle), SVR C (open diamond), and HSVR (solid square) for the test samples. The solid, dashed, and dotted lines represent the HSVR regression of the data, 95% confidence intervals for the HSVR regression, and 95% confidence intervals for the prediction, respectively.

The predicted values by SVR A, SVR B, and SVR C are in moderate agreement with the observed values for those test molecules depicted by Figure 3, which shows the scatter plot of observed versus the log *P*_app_ predictions by SVR A, SVR B, SVR C, and HSVR for those samples in the test set. The MAE values generated by SVR A, SVR B, and SVR C increase from 0.28, 0.17, and 0.17 in the training set to 0.42, 0.35, and 0.39 in the test set, respectively (Table 3). RMSE along with the other statistic values also reveal deteriorating performances of these models in SVRE from the training set to the test set (Table 2 and Table 3). Moreover, the *q*^2^ values produced by SVR A, SVR B, and SVR C were 0.50, 0.58, and 0.60 in the test set, respectively, which are much less than their *r*^2^ counterparts in the training set.

**Figure 4:**
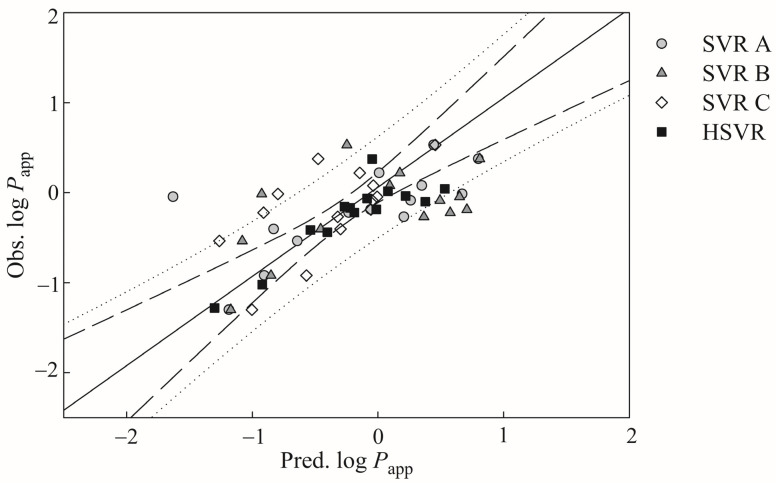
Observed log *P*_app_ versus the log *P*_app_ predicted by SVR A (gray circle), SVR B (gray triangle), SVR C (open diamond), and HSVR (solid square) for the outlier samples. The solid, dashed, and dotted lines represent the HSVR regression of the data, 95% confidence intervals for the HSVR regression, and 95% confidence intervals for the prediction, respectively.

The prediction performances of those SVR models in the SVRE were significantly decreased when applied to those samples in the outlier set as suggested by the statistical metrics listed in Table 4. For example, SVR A, SVR B, and SVR C yielded the $q_{F2}^{2}$ values of −0.18, −0.41, and 0.16, respectively, which are substantially smaller than the *r*^2^ values in the training set and the $q_{F2}^{2}$ values in the test set (Table 2 and Table 3). Furthermore, the distances between the points and the regression line in the outlier set were much greater than those in the training set shown in Figure 4. As such, it can be asserted that those three models in the SVRE are vulnerable to the outliers that, actually, are not uncommon for most predictive models [100].

### 3.3. HSVR Model

The HSVR model was generated by the regression of SVRE according to the predictions of all molecules and statistical assessments in the training set (Table S1 and Table 2), and its runtime parameters are recorded in Table S2. HSVR commonly predicted better than SVR A, SVR B, and SVR C for the samples in the training set, as demonstrated by Figure 2, from which it can be noticed that most of predictions by HSVR lie in the range between the largest and the smallest ones predicted by those models in the SVRE. HSVR can improve the predictions in some cases. For instance, the prediction of compound **101** (omeprazole) by HSVR yielded an absolute residual of 0.02, whereas SVR A, SVR B, and SVR C produced the absolute errors of 0.34, 1.10, and 0.18, respectively (Table S1). In addition, HSVR produced the highest *r*^2^ (0.91) and $q_{\mathrm{CV}}^{2}$(0.81) and the lowest Δ_Max_ (0.98), MAE (0.10), *s* (MAE), and RMSE (0.20) values when compared with those models in the SVRE, suggesting that HSVR statistically performed better SVR A, SVR B, and SVR C in the training set. Furthermore, HSVR gave rise to a $\langle r_{s}^{2}\rangle$ value of 0.03, indicating that it is least possible that HSVR was created by chance correlation [87].

When applied to the test molecules, marginal performance deteriorations can be found for HSVR. For example, *s* increased from 0.18 in the training set to 0.20 in the test set (Table 2 and Table 3). However, Δ_Max_ dropped from 0.98 in the training set to 0.72 in the test set. HSVR still executed better than SVR A, SVR B, and SVR C in the test set as shown in Figure 3. The other statistical parameters listed in Table 3 also assert the performance dominance of HSVR. For instance, the *q*^2^ values were 0.50, 0.58, 0.60, and 0.75 generated by SVR A, SVR B, SVR C, and HSVR, respectively. Similarly, HSVR also produced smaller absolute deviations than its counterparts in the SVRE in the test set. For example, the absolute residuals of compound 36 (clozapine) were 0.35, 0.54, 0.35, and 0.03 yielded by SVR A, SVR B, SVR C, and HSVR, respectively (Table S1). HSVR generally produced consistent and small deviations in both training and test sets as asserted by those parameters listed in Table 2 and Table 3 in comparison with its counterparts in the SVRE. More importantly, the HSVR model generated the largest *q*^2^ (0.75) in the test set and the smallest difference between *r*^2^ and $q_{\mathrm{CV}}^{2}$ (0.10), suggesting that it is less likely that HSVR model was over-trained or over-fitted.

HSVR even displayed better performance than the SVR models in the ensemble in the outlier set as depicted by those statistical assessments listed in Table 4. The HSVR model generated the largest $q^{2}$ value (0.76) and yet SVR A, SVR B, and SVR C yielded 0.45, 0.36, and 0.40, respectively. The superiority of HSVR in the outlier set can also be assured by the other statistical parameters, which is mainly due to the broader application domain of HSVR when compared with its counterparts in the ensemble. That robust HSVR feature makes it more utilizable in practical applications [101].

### 3.4. Predictive Evaluations

**Figure 5:**
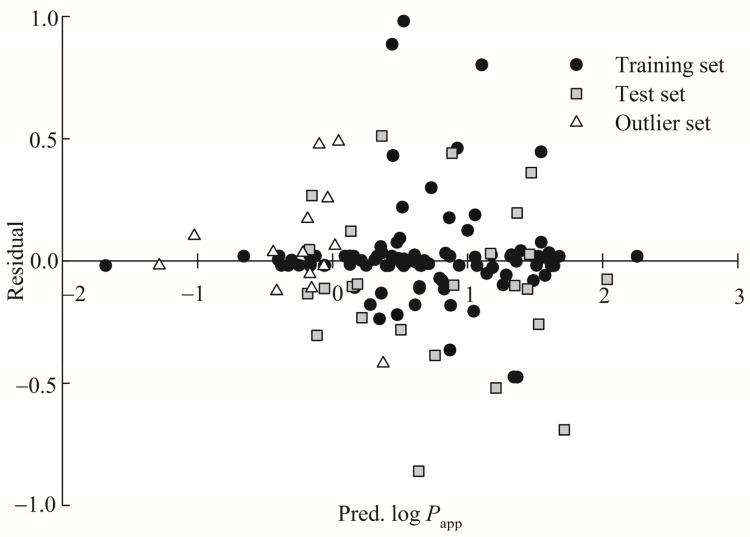
Residual versus the log *P*_app_ prediction by HSVR in the training set (solid circle), test set (gray square), and outlier set (open triangle).

The scatter plot of residual versus the log *P*_app_ prediction by HSVR for the training, test, and outlier samples is shown in Figure 5, from which it can be found that the residuals are commonly situated on both sides of x-axis along with the prediction range in those three datasets, suggesting that it is least likely that systematic error is associated with HSVR. Additionally, the training set, test set, and outlier set had the average residuals of 0.02, −0.13, and 0.06, respectively (Table S1), denoting that there is no biased prediction by HSVR.

Table 5 lists the results when the developed HSVR model was further subjected to the most stringent validation criteria collectively recommended by Golbraikh et al. [82], Ojha et al. [96], Roy et al. [98], and Chirico and Gramatica [89] in the three datasets (Equations (15)–(21)). It can be observed that HSVR completely met those proposed validation requirements in addition to the fact that HSVR exhibited a similar degrees of performance in the training set, test set, and outlier set. As such, it can be asserted that HSVR is an extremely accurate and predictive theoretical model.

### 3.5. Mock test

**Figure 6:**
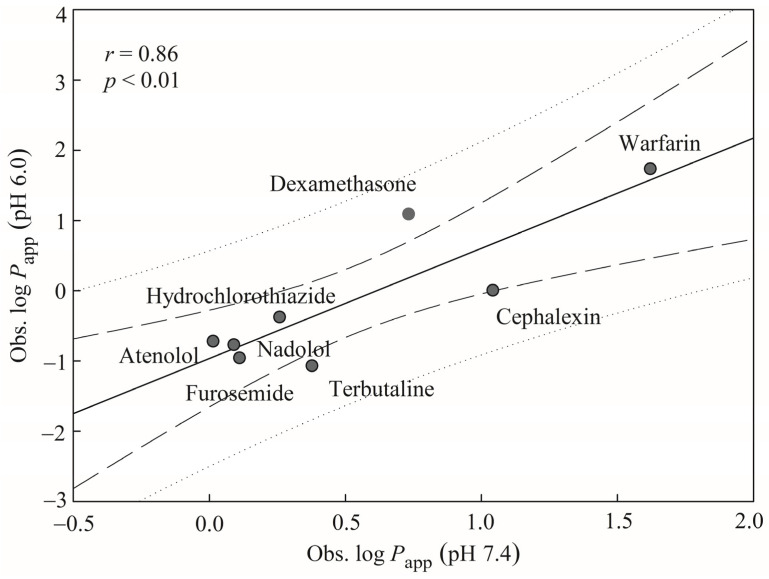
Observed log *P*_app_ at pH 7.4 versus observed log *P*_app_ at pH 6.0 for the common drugs in the mock test. The solid, dashed, and dotted lines represent the mock test regression of the observed data, 95% confidence interval for the mock test regression, and 95% confidence interval for the observation, respectively.

To verify the practical applicability of the generated HSVR model, this model was applied to those drugs measured by Yamashita et al. [30]. There were eight compounds commonly adopted by this study and Yamashita et al., furnishing a sound way to calibrate the challenging system. However, Yamashita et al. assayed the *P*_app_ values at pH 6.0, instead of pH 7.4 used by those compounds collected in this study, suggesting that some *P*_app_ variations can be resulted from both systems (vide supra). These discrepancies make those drugs assayed by Yamashita et al. not appropriate as the second external dataset or the test set because those validation criteria listed in Table 5 cannot be applied to those drugs. The relationship between both different experimental conditions was initially constructed for those eight common compounds, and the resulting scatter plot is exhibited in Figure 6, from which it can be found that both assay systems were reasonably correlated with each other with an *r* value of 0.86), suggesting that this HSVR can be adopted to predict those novel compounds measured by Yamashita et al.

**Figure 7:**
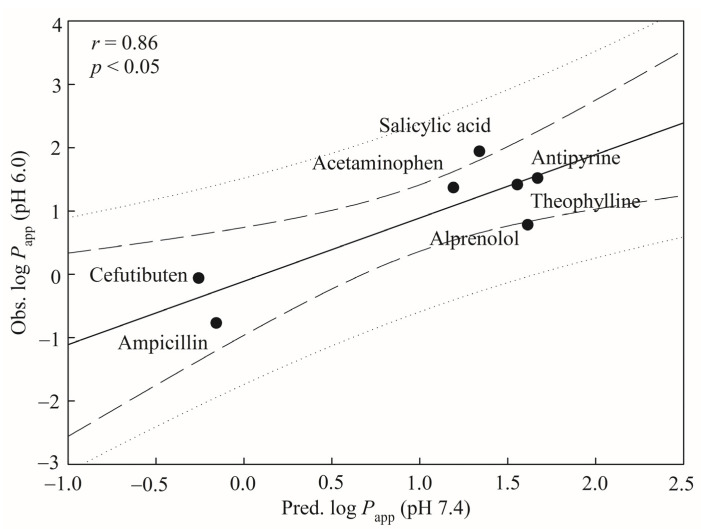
Predicted log *P*_app_ at pH 7.4 versus observed log *P*_app_ at pH 6.0 by the HSVR model for the drugs in the mock test. The solid, dashed, and dotted lines represent the HSVR regression data, 95% confidence interval for the HSVR regression, and 95% confidence interval for prediction, respectively.

Figure 7 shows the predicted results of seven novel drugs in the mock test. The correlation coefficient *r* value between the predicted log *P*_app_ (pH 7.4) and observed log *P*_app_ (pH 6.0) was 0.86, suggesting that the HSVR model can nearly reproduce the experimental results. In addition, the produced *p*-value was <0.05. This mock test ensured the predictive ability of generated HSVR when applied to the novel compounds with different experimental conditions.

### 3.6. Classification

It is of interest to verify the qualitative predictivity of HSVR, since a number of qualitative models have been published [25,102]. Accordingly, compounds enlisted in this study were classified as Caco-2 permeable (Caco-2^+^) and Caco-2 impermeable (Caco-2^‒^) based on the threshold value of *P*_app_ (8 × 10 ^‒6^ cm/s) as suggested [25,102]. Initially, the confusion matrix was constructed (Table S3), and the Cooper statistics and Kubat’s G-mean [103] (Table S4) were employed to qualitatively evaluate the predictivity of HSVR. The results were also compared with predictions made by *admetSAR* [104] (available at website: http://lmmd.ecust.edu.cn/admetsar2/), since *admetSAR* has been adopted by DrugBank (available at: https://go.drugbank.com/) to qualitatively predict Caco-2 permeability. The results are listed in Table 6, from which it can be asserted that HSVR outperformed *admetSAR* in every aspect. For instance, the parameter accuracy was 93.1% produced by HSVR, which is substantially higher than that generated by *admetSAR* (50.7%). The metric MCC is the most distinction between HSVR and *admetSAR* (85.0% vs. −8.0%). Thus, it can be asserted that HSVR is also an accurate and predictive qualitative predictive model.

## 4. Discussion

Caco-2 has been commonly adopted to predict the intestinal permeability in the process of drug discovery because of its morphological and functional similarity with human enterocytes [105]. The mechanism of Caco-2 permeation is rather complex, since it can take place through passive diffusion, which can go through the paracellular and transcellular routes and active transport. The passive diffusion is predominately governed by the concentration gradient, and most hydrophilic drugs prefer to penetrate between cells in a paracellular fashion, whereas hydrophobic drugs are inclined to get across the cells via the transcellular route. Drugs that can permeate the Caco-2 cells by the active transport can interact with the influx and/or efflux transporters expressed on the cell surface [106]. As such, Caco-2 permeability is affected by some physicochemical and physiological properties [106].

**Figure 8:**
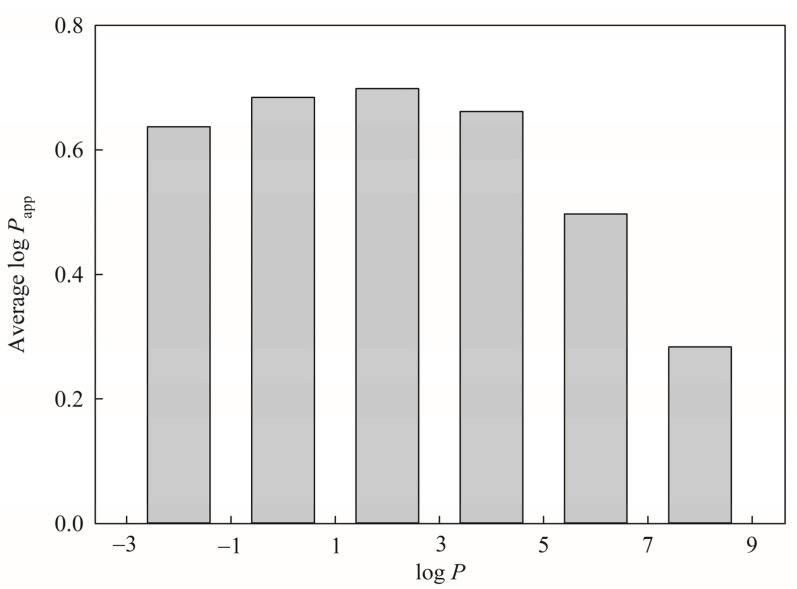
Histogram of average log *P*_app_ versus the distribution of log *P*.

Hydrophobicity or lipophilicity plays an important role in passive diffusion through membranes as well as the drug–receptor interactions [17,107,108]. In addition, hydrophobicity, which can represent by the *n*-octanol‒water partition coefficient, *viz*. log *P*, is also an important factor affecting the interaction between the molecules and the target protein, since more lipophilic molecules tend to have stronger interactions with both target protein and biological membrane. Therefore, the very lipophilic molecules have poor oral absorption from the stomach [107,109]. Polar and hydrophobic drug must penetrate through the Caco-2 cell membrane [17,110]. In addition, it has been observed that log *P*, hydrogen bond propensity, weight, and volume are closely related with *P*_app_ [43]. As such, log *P* was adopted in this study (Table 1), which is consistent with the fact that numerous published in silico models to predict intestinal absorption, PAMPA permeability [1,111], and Caco-2 permeability also have employed this descriptor [40,112,113,114]. It can be observed from Figure 8, which displays the average log *P*_app_ for each histogram bin of log *P* for all molecules included in this investigation, that log *P*_app_ increased with log *P* value initially and then decreased afterward, leading to a seemingly bilinear relationship between log *P*_app_ and log *P*. This perplexing dependency can be realized by the fact that the more hydrophobic solutes can easier approach the lipid bilayer to penetrate the membrane. The opposite relationship between hydrophobicity and permeability will be resulted when the solutes are too hydrophobic due to stronger attractions between solutes and the membrane as well as stronger repulsive forces from the solvent molecules upon the entrance to the solvent environment that can be illustrated by the PAMPA permeability [1,115,116]. Complexity can be even profound when taking into account the fact that P-gp and BCRP, which are efflux transporters in Caco-2 (vide supra), can interact with substrates by hydrophobicity [117], subsequently leading to a low correlation between log *P*_app_ and log *P* (*r* = 0.15).

It has been observed that the number of aromatic rings (*n*_Ar_) has a positive correlation with log *P* with an *r* value of 0.67 [118], suggesting that a predictive model can be over-trained once both log *P* and *n*_Ar_ are adopted simultaneously. However, this issue was not concerned in this study, since only SVR C adopted this descriptor, whereas SVR A and SVR B included log *P* (Table 1). In addition, the aromatic ring is a non-polar group, which can enhance the hydrophobicity [52] and increase the passive diffusion [119,120]. In addition, aromatic ring moieties have been implicated in P-gp substrate recognition and efflux modulation [53], leading to the fact that *n*_Ar_ can be an important factor in P-gp modulation action [121] and BCRP‒substrate interactions [122]. As such, *n*_Ar_ plays a complex role in both passive diffusion and active transport in Caco-2 permeability.

It has been recognized that both PSA and *µ* are associated with passive diffusion [37,123,124,125]. In addition, these descriptors have been adopted by published in silico Caco-2 permeability models [37,45,46,47,48,49,126,127,128]. It has been reported in the PAMPA permeability study that larger PSA, *µ*, and polarity can enhance the solute‒solute and solute‒solvent interactions, which, in turn, require more desolvation energy when the solutes penetrate through the lipophilic membrane to the donor compartment [123,129,130,131,132], and conversely decrease the passive diffusion [1], consequently, making permeability less favorable. Therefore, it has been shown that PSA has a negative impact in the permeation rate [133,134]. In addition, Joung et al. have indicated that PSA shows an important role in distinguishing the P-gp substrate from the non-substrates [135]. Accordingly, PSA and *µ* were adopted in this study due to their pivotal roles in Caco-2 permeability.

**Figure 9:**
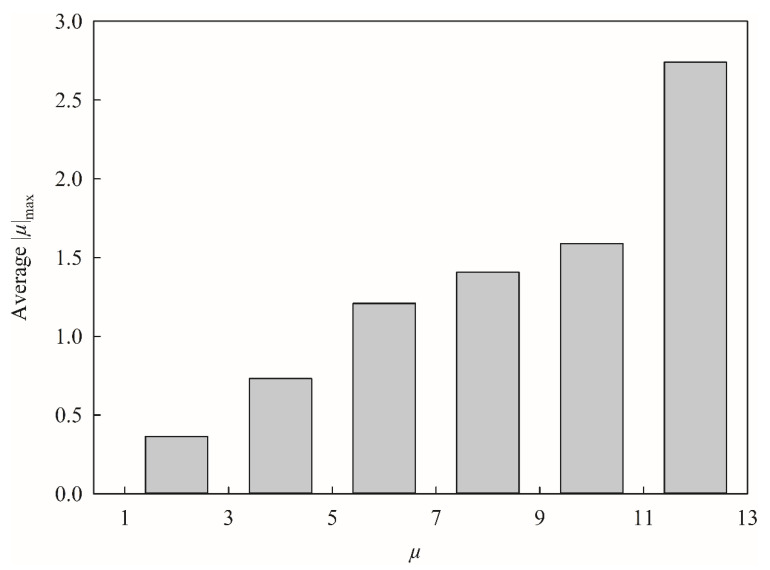
Histogram of average |*µ*|_max_ versus the distribution of *µ*.

It is seemingly unusual to include the descriptor |*µ*|_max_, which is the absolute maximum component of the molecular dipole, in this study, since it has never been employed by any published model before. This inconsistency actually can be manifested by Figure 9, which displays the average |*µ*|_max_ for each histogram bin of *µ*, that the larger *µ*, the larger |*µ*|_max_, suggesting that they were positively correlated with each other. In addition, *µ* was recruited by SVR A and SVR C, whereas |*µ*|_max_ was enlisted by SVR B only, suggesting that it is less likely to produce an over-trained HSVR, since no single model adopted both two descriptors simultaneously. More importantly, the empirical observation has revealed that HSVR including these selections executed better than the others (data not shown) plausibly because of the descriptor‒descriptor interaction [1]. Any other traditional linear or machine learning-based QSAR schemes, conversely, cannot properly render such contradictory descriptor selections.

It has been reported that the molecular size of the solute molecule is of critical importance in the diffusivity of the biological membrane [37,125,136], and the intestinal absorption can decrease with the increase of molecular size [137]. Furthermore, the molecular size also affects passive diffusion through membranes [138,139] and active transport through the P-gp‒substrate interactions [121,138]. Molecular size can be represented by a number of descriptors such α, *n*_Ring_, *V*_m_, and *n*_rot_ [140,141,142], which were adopted in the investigation and negatively associated with log *P*_app_ (Table 1). Conversely, Fujiwara et al. adopted the descriptor molecule weight (MW) to develop a theoretical Caco-2 permeability model [37], whereas MW was not included in this study. This discrepancy can be realized by the fact that α was highly correlated with MW with an *r* value of 0.98 for all molecules enlisted in this study, suggesting that it is plausible to replace MW by α in order not to produce an over-trained model. In addition, it has been observed that α is positively correlated to log *P* [143] and is highly associated with absorption [50].

**Figure 10:**
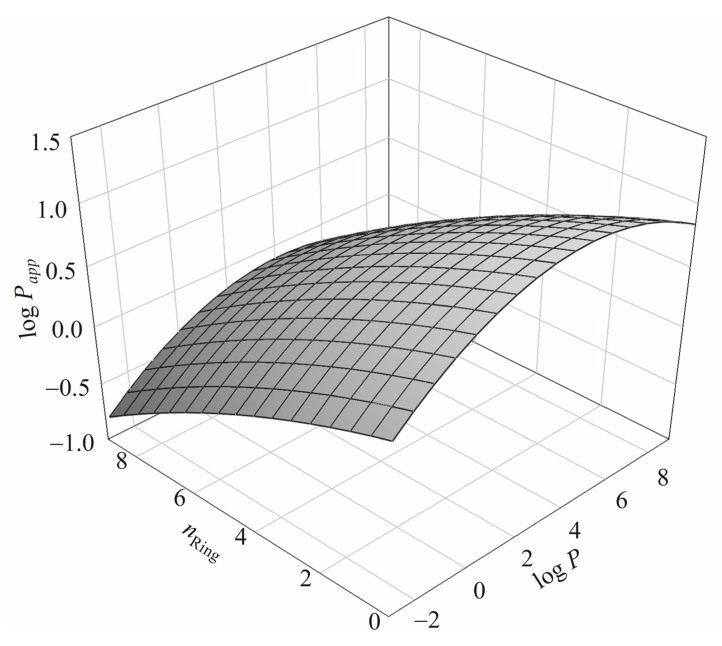
The relationship among log *P*_app_, *n*_Ring_, and log *P* in 3D presentation.

The descriptor *n*_Ring_, which is reportedly related to molecular size [136,141], has never been adopted by any published Caco-2 permeability predictive model and yet was selected by SVR C (Table 1). This disagreement can be recognized by the fact that *n*_Ring_ was greatly correlated with α with an *r* value of 0.78 for all molecules recruited in this study. As such, it is plausible to expect that both *n*_Ring_ and α play similar roles in Caco-2 permeability. The relationship among log *P*_app_, *n*_Ring_, and log *P* can be further perplexing as illustrated by Figure 10, which shows the 3D plot of log *P*_app_, *n*_Ring_, and log *P*. The relationship between *n*_Ring_ and log *P* has been detailed by Pham-The et al. [125].

It has been observed that *V*_m_ plays an important role in passive absorption [9,144,145] and it is adopted by a published Caco-2 permeability model [146] as well as in this study. It has been observed in the rat that fewer rotatable bonds, viz. smaller *n*_rot_, can lead to better oral bioavailability, and *n*_rot_ can also exert a positive effect on the permeation rate [133,143], since more rigid molecules will have smaller *n*_rot_ values that, in turn, can enhance permeability [125]. Furthermore, *n*_rot_ is of importance in intestinal absorption [147], since increased *n*_rot_ can reduce the permeability [133]. Furthermore, a number of published membrane permeability models have also employed the descriptor CSA, which is another feature associated with molecular size and also plays a pivotal role in membrane permeability [70,71]. However, *n*_rot_ was greatly associated with CSA with an *r* value of 0.80 for all molecules enrolled in this investigation, suggesting that using *n*_rot_ in lieu of CSA without producing the over-trained model is plausible. Li et al. also have found that *n*_rot_ is another feature to discriminate P-gp substrates from non-substrates [148]. As such, it is of necessity to recruit *n*_rot_ in model development to properly render Caco-2 permeability as suggested [71,72].

Hydrogen bonding potential, which can be expressed by HBD and HBA, is another important factor in determining the solute–solvent interactions [37], and it is the main contributor for the passive diffusion [143]. It has been observed that Caco-2 permeability is a function of HBD and/or HBA, since more permeable solutes tend to have smaller HBD and/or HBA [130,131,149]. Between HBD and HBA, HBD seemingly shows a more profound effect on Caco-2 permeability as compared with HBA [150] as manifested by the fact that several published in silico models have selected HBD to predict Caco-2 permeability instead of HBA [35,42]. Mechanistically, HBD is one of the features associated with P-gp‒substrate interactions [148,151]. In addition to efflux transport, HDB is one of the features linked to substrate binding with OATP2B1 [7] as well as PepT1 [152]. Thus, it is of necessity to include in Caco-2 predictive models to take into consideration the passive diffusion as well as the active influx/efflux transport.

**Figure 11:**
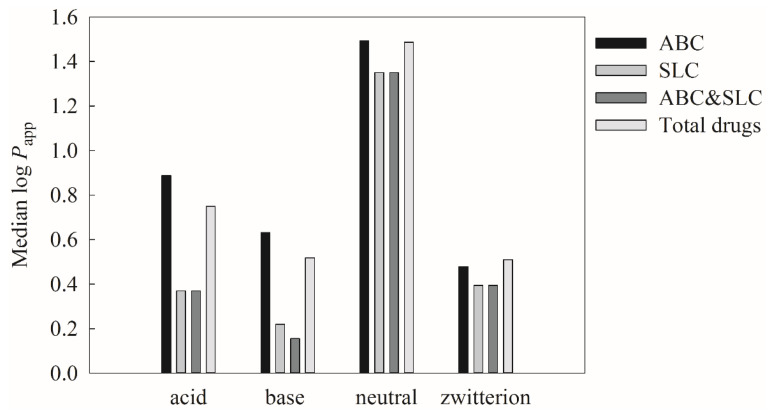
The histogram represents the log *P*_app_ versus the molecules belong to ATP-binding cassette (ABC) substrate, solute carrier (SLC) substrate, both ABC and SLC substrate, and total drugs in the acid class, base class, neutral class, and zwitterion class, respectively.

The descriptor p*K*_a(Max)_ was selected in this study due to the fact that higher p*K*_a(Max)_ can lead to the lower ionized form of drugs in the donor compartment, which, in turn, can increase the penetration through hydrophobic membrane [153]. Furthermore, it has been recognized that neutral compounds can have higher membrane permeability than the other ion classes [154]. Accordingly, all molecules included in this investigation were categorized into different ion classes based on their p*K*_a_ values. In addition, ABC and/or SLC substrates were also identified based on the drug information retrieved from DrugBank to understand if the dependence of ion class can be varied by their ion classes. It can be found from Figure 11, which displays the histograms of median log *P*_app_ versus all molecules, ABC substrates, SLC substrates, as well as ABC and SLC substrates for four different ion classes, that the median log *P*_app_ values of neutral compounds are substantially larger than the others, suggesting that neutral compounds exhibit higher Caco-2 permeability regardless of active transporter substrate classes, viz. influx transporter or efflux transporter. This observation actually is very similar to the PAMPA permeability, since the ionized compounds will demand larger desolvation energies, which, in turn, can hinder their penetration [134].

Initially, numerous efforts were made in attempting to build assorted 2-QSAR models by employing the partial least square (PLS) scheme, and yet no productive models were produced (data not shown) [1]. This challenge can be realized by the fact that the correlations between the designated descriptors and log *P*_app_ for all molecules included in this investigation were small, and the largest absolute maximum *r* was only 0.56 between PSA and log *P*_app_ (Table 1), signifying the high non-linearity between them. More significantly, the substantial difference in 2-QSAR development between the passive diffusion, viz. the PAMPA system, and Caco-2 permeability can be greatly attributed to the complex active (influx and efflux) transport. Thus, it is extremely difficult, if not absolutely impossible, to derive a linear Cacao-2 permeability QSAR model. Conversely, the accurate and predictive HSVR model can properly render such non-linear dependence of log *P*_app_ on descriptors.

## 5. Conclusions

Intestinal permeability is one of the important ADME/Tox metrics that should be addressed in the process of drug discovery and development. The Caco-2 system has been frequently used as a surrogate to preliminarily investigate the intestinal absorption. An in silico model can be a useful approach to predict Caco-2 permeability in assisting drug discovery and development. However, Caco-2 permeability can occur through passive diffusion and active transport, leading to a complex process. Therefore, it is of necessity to include different descriptor combinations and diverse relationships to address these variations in distinct mechanisms. The innovative machine learning-based HSVR scheme, which possesses the superior features of a local model (greater predictivity) and a global model (larger coverage of the application domain), was employed in this study to construct a theoretical model to predict the Caco-2 permeability. The generated HSVR models unveiled great prediction accuracy for the training, test, and outlier samples. When challenged by a group of drugs assayed at different experimental conditions, the developed HSVR model also executed equivalently well. In addition, HSVR showed excellent qualitative performance in recognizing Caco-2 permeable and impermeable compounds, and the selected descriptors can completely justify the diverse mechanisms related to the passive diffusion and active transport. Thus, it can be assured that this HSVR model can be useful to accurately and swiftly predict the Caco-2 permeability of novel compounds in order to assist drug discovery and development.

## Figures and Tables

**Table 1:** The list of ensemble support vector regression (SVR) models and their descriptors, the correlation coefficient (*r*) with *P*_app_, and their descriptions.

Descriptor	SVR A	SVR B	SVR C	*r*	Description
log *P*	X ^†^	X		0.15	Logarithm of the *n*-octanol‒water partition coefficient
*n*_Ar_			X	−0.07	Number of aromatic rings
PSA	X	X		−0.56	Polar surface area
*µ*		X		−0.27	Dipole moment
|*µ*|_max_	X		X	−0.08	The maximum dipole component
α	X			−0.34	Sum of atomic polarizabilities over all the molecule atoms
*n*_Ring_			X	−0.31	Number of rings
*V*_m_			X	−0.35	Molecular volume
*n*_Rot_			X	−0.21	Number of rotatable bonds in a molecule
HBD		X	X	−0.40	Number of hydrogen-bond donors
p*K*_a(Max)_	X		X	−0.13	The maximum p*K*_a_ for a molecule
ion class		X		N/A^‡^	Four classes are separated by the p*K*_a_ of molecules

^†^ Selected. ^‡^ Not applicable.

**Table 2:** Statistic metrics including *r*^2^, ∆_Max_, mean absolute error (MEA), *s*, root mean square error (RMSE), $q_{\mathrm{CV}}^{2}$, and $\langle r_{s}^{2}\rangle$ assessed by support vector regression (SVR) A, SVR B, SVR C, and hierarchical support vector regression (HSVR) in the training set.

Statistic Metrics	SVR A	SVR B	SVR C	HSVR
*r* ^2^	0.69	0.77	0.76	0.91
Δ_Max_	1.31	1.19	1.66	0.98
MAE	0.28	0.17	0.17	0.1
*s*	0.25	0.28	0.29	0.18
RMSE	0.38	0.32	0.33	0.2
$q_{\mathrm{CV}}^{2}$	0.16	0.19	0.21	0.81
$\langle r_{s}^{2}\rangle$	0.05	0.03	0.03	0.03

**Table 3:** Statistic metrics including $q_{}^{2}$, $q_{F1}^{2}$, $q_{F2}^{2}$, $q_{F3}^{2}$ *CCC*, ∆_Max_, MAE, *s*, and RMSE assessed by SVR A, SVR B, SVR C, and HSVE in the test set.

Statistic Metrics	SVR A	SVR B	SVR C	HSVR
$q_{}^{2}$	0.50	0.58	0.60	0.75
$q_{F1}^{2}$	0.42	0.58	0.59	0.71
$q_{F2}^{2}$	0.41	0.57	0.59	0.71
$q_{F3}^{2}$	0.30	0.50	0.50	0.70
*CCC*	0.62	0.74	0.77	0.85
Δ_Max_	1.27	1.06	0.88	0.72
MAE	0.42	0.35	0.39	0.33
*s*	0.35	0.31	0.23	0.20
RMSE	0.54	0.46	0.45	0.38

**Table 4:** Statistic metrics including *q*^2^, $q_{F1}^{2}$, $q_{F2}^{2}$, $q_{F3}^{2}$, *CCC*, ∆_Max_, MAE, *s*, and RMSE assessed by SVR A, SVR B, SVR C, and HSVE in the outlier set.

Statistic Metrics	SVR A	SVR B	SVR C	HSVR
*q* ^2^	0.45	0.36	0.40	0.76
$q_{F1}^{2}$	0.75	0.70	0.82	0.95
$q_{F2}^{2}$	−0.18	−0.41	0.16	0.76
$q_{F3}^{2}$	0.39	0.27	0.56	0.87
*CCC*	0.49	0.56	0.58	0.87
Δ_Max_	1.58	0.91	0.82	0.49
MAE	0.35	0.47	0.16	0.17
*s*	0.41	0.34	0.56	0.17
RMSE	0.52	0.57	0.58	0.24

**Table 5:** Validation verification of HSVR based on prediction performance of the training, test, and outlier samples.

Validation Verification	Training Set	Test Set	Outlier Set
$r_{0}^{2}$	0.91	0.75	0.75
k	1.01	0.86	0.93
${r^{\prime }}_{0}^{2}$	0.91	0.68	0.71
$r_{m}^{2}$	0.84	0.71	0.68
${r_{\prime }}_{m}^{2}$	0.91	0.75	0.76
$\langle r_{m}^{2}\rangle$	0.87	0.73	0.72
$\Delta r_{m}^{2}$	0.06	0.04	0.08
$r_{}^{2}\geq 0.70$	X ^†^	X	X
Equation (15)	X	X	X
Equation (16)	X	N/A	N/A
Equation (17)	X	X	X
Equation (18)	X	X	X
Equation (19)	X	X	X
Equation (20)	X	X	X
Equation (21)	N/A ^‡^	X	X

^†^ Fulfilled; ^‡^ Not applicable.

**Table 6:** Statistical parameters of qualitative predictions by HSVR and *admetSAR*.

Statistical Parameters	HSVR	*admetSAR*
Se	90.0%	32.0%
Sp	94.7%	60.6%
Acc	93.1%	50.7%
PP	90.0%	30.2%
NP	94.7%	62.6%
MCC	85.0%	−8.0%
*G*-mean	92.3%	44.1%
*F-measure*	90.0%	31.1%
*κ*	85.0%	−8.0%

## Data Availability

Not applicable.

## Supplementary Materials

The following are available online at https://www.mdpi.com/1999-4923/13/2/174/s1, Table S1. Selected compounds for this study; their names, SMILES strings, CAS numbers, and observed log *P*_app_ values; their predicted values by SVR A, SVR B, SVR C, and HSVR; data partitions; and references; Table S2. Optimal runtime parameters for the SVR models; Table S3. Confusion matrix for the qualitative predictive model; Table S4. The Cooper statistics and Kubat’s G-mean calculated from the confusion matrix. Optimal runtime parameters for the SVR models; Figure S1. Histograms of: (A) log *P*app, (B) molecular weight (MW), (C) surface area (SA), (D) polar surface area (PSA), (E) number of hydrogen bond acceptor (HBA), (F) number of hydrogen bond donor (HBD), (G) and the *n*-octanol-water partition coefficient (log *P*) in the training set, test set, and outlier set.
