# Supplementary material for: Development of a Hierarchical Support Vector Regression-Based In Silico Model for Caco-2 Permeability
Source: Pharmaceutics. 2021 Jan 28;13(2):174. doi: 10.3390/pharmaceutics13020174 (PMC7911528; doi:10.3390/pharmaceutics13020174)
Supplement: Supplementary file 1 [file pharmaceutics-13-00174-s001.pdf]

pharmaceutics

Supplementary Materials: Development of a Hierarchical Support Vector Regression-Based In Silico Model for Caco-2 Permeability

Giang Huong Ta [1], Cin-Syong Jhang [1], Ching-Feng Weng [2] and Max K. Leong [1,*]

Citation: Ta, G.H.; Jhang, C.-S.; Weng, C.-F.; Leong, M.K. Development of a Hierarchical Support Vector Regression-Based In Silico Model for Caco-2 Permeability. *Pharmaceutics* **2021**, *13*, 174. https://doi.org/10.3390/pharmaceutics13020174

Academic Editor: Maria Isabel Gonzalez-Alvarez
Received: 27 December 2020
Accepted: 21 January 2021
Published: 27 January 2021

Publisher's Note: MDPI stays neutral with regard to jurisdictional claims in published maps and institutional affiliations.

Copyright: © 2021 by the authors. Licensee MDPI, Basel, Switzerland. This article is an open access article distributed under the terms and conditions of the Creative Commons Attribution (CC BY) license (http://creativecommons.org/licenses/by/4.0/).

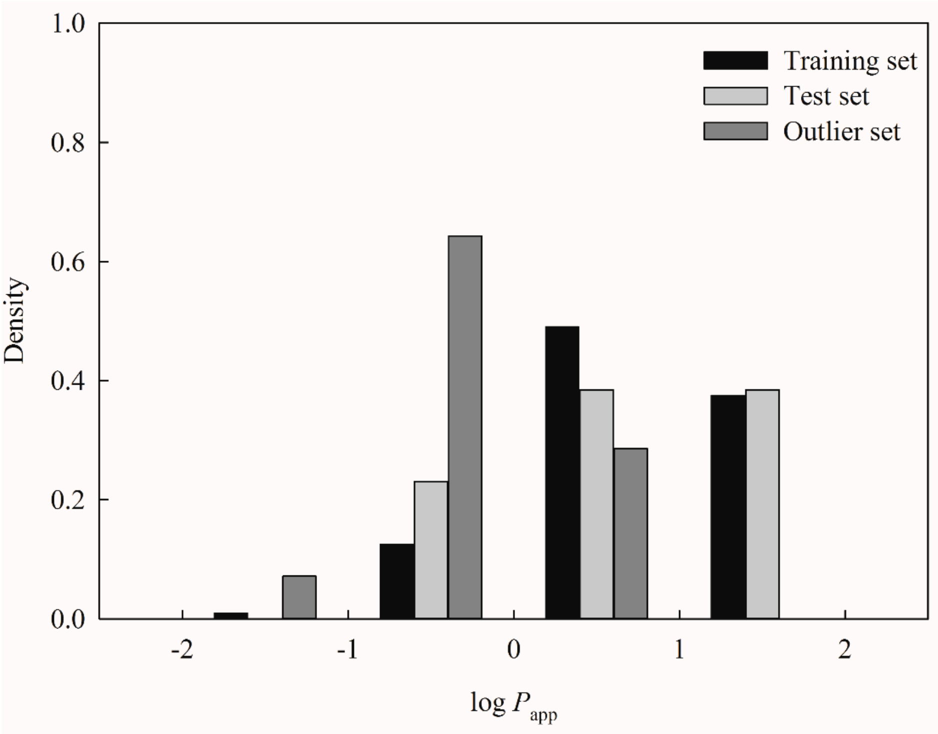

(A)

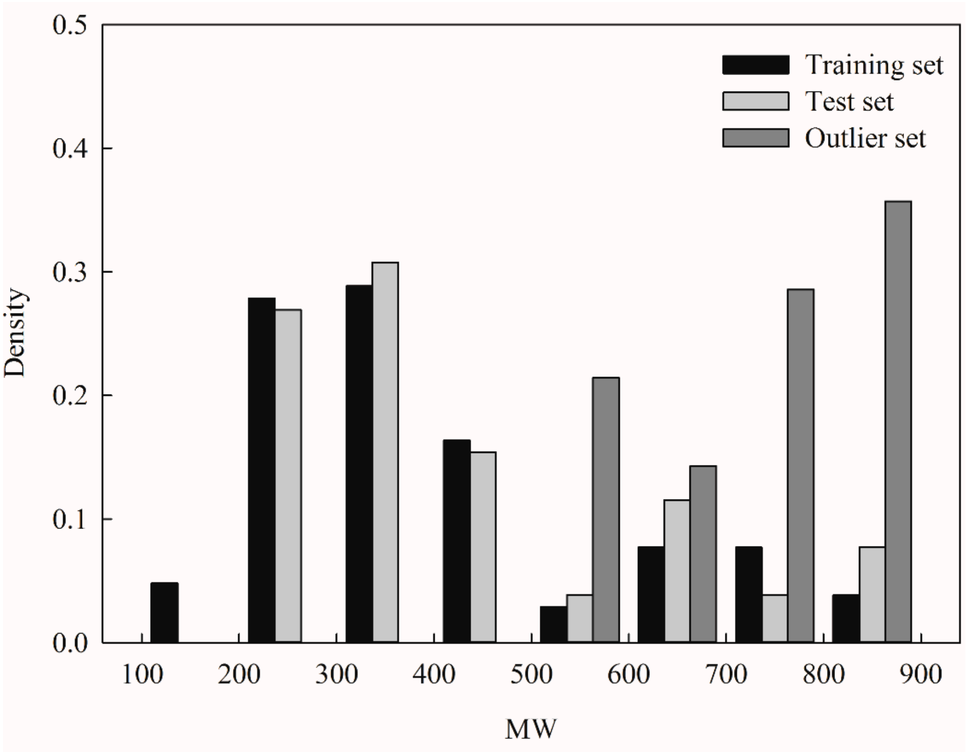
Training set
Test set
Outlier set
Density
0.0
0.1
0.2
0.3
0.4
0.5
100
200
300
400
500
600
700
800
900
MW

(B)

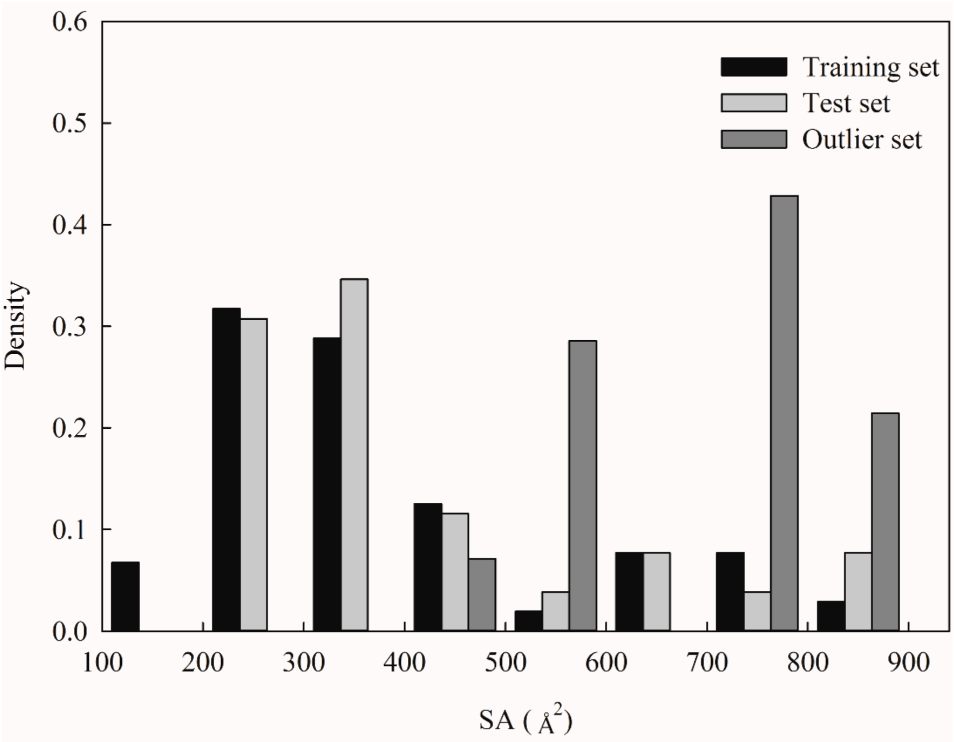
Training set
Test set
Outlier set
Density
0.0
0.1
0.2
0.3
0.4
0.5
0.6
100
200
300
400
500
600
700
800
900
SA (Å2)

(C)

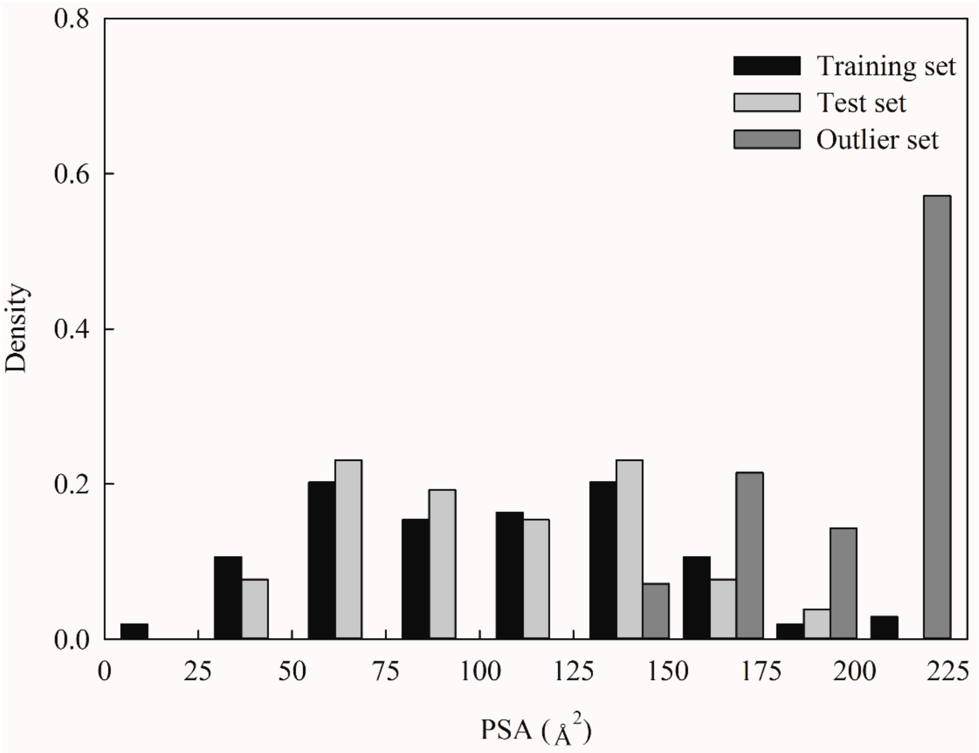
Training set
Test set
Outlier set
Density
0.0
0.2
0.4
0.6
0.8
0
25
50
75
100
125
150
175
200
225
PSA (Å²)

(D)

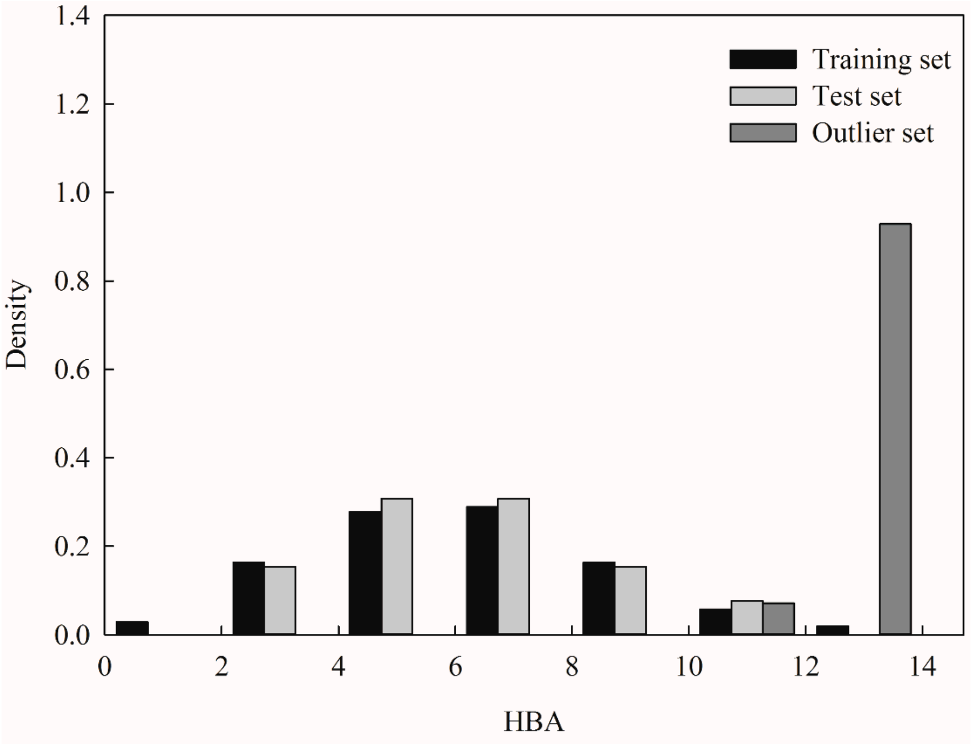
Training set
Test set
Outlier set
Density
0.0
0.2
0.4
0.6
0.8
1.0
1.2
1.4
0
2
4
6
8
10
12
14
HBA

(E)

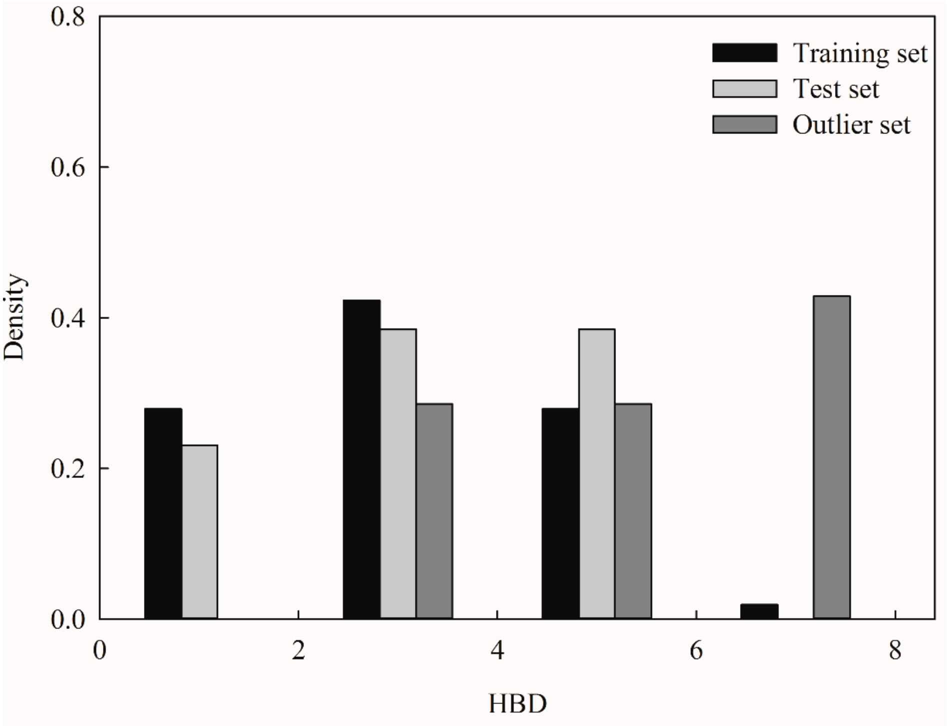

(F)

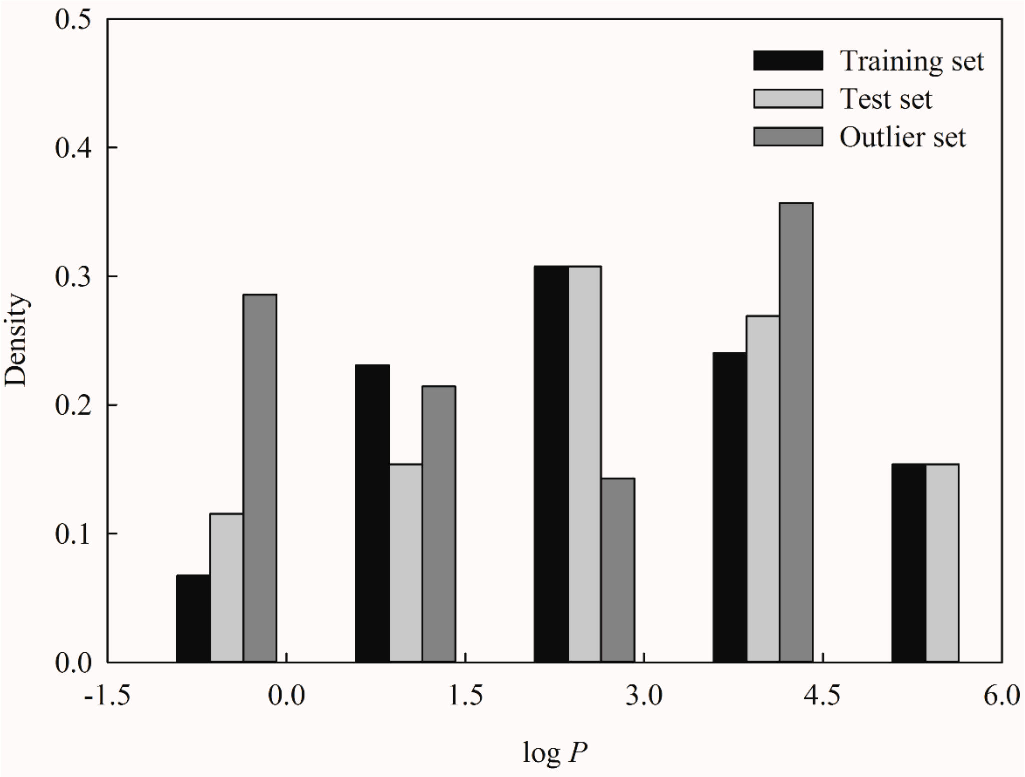

(G)

Figure S1. Histogram representation of the distributions of various descriptors for all molecules in the training set, test set and outlier set. (**A**) log *P*app, (**B**) molecular weight (MW), (**C**) surface area (SA), (**D**) polar surface area (PSA), (**E**) number of hydrogen bond acceptor (HBA), (**F**) number of hydrogen bond donor (HBD), (**G**) and the *n*-octanol-water partition coefficient (log *P*) in the training set, test set and outlier set.

Table S1. Compound source for this study, their names, IUPAC names, CAS numbers, , SMILES strings, observed log Papp values, and predicted values by SVR A, SVR B, SVC, and HSVR, data partition, and reference.

No.	Molecules	IUPACz	CAS	Smiles	Obs. Log P_{app}	SVR A	|Δ|	SVR B	|Δ|	SVR C	|Δ|	HSVR	|Δ|	Set†	References
**1**	Acetaminophen	*N*-(4-hydroxyphenyl)acetamide	103-90-2	O=C(N([H])C1=C([H])C([H])=C(O[H])C([H])=C1[H])C([H])([H])[H]	-0.15	0.10	0.25	-0.40	0.25	-0.15	0.00	-0.19	0.04	T	Lozoya-Agullo *et al.* 2015; Stewart *et al.* 1995; Deng *et al.* 2007
**2**	Aloin	(*S*)-1,8-dihydroxy-3-(hydroxymethyl)-10-((2*S*,3*R*,4R,5*S*,6*R*)-3,4,5-trihydroxy-6-(hydroxymethyl)tetrahydro-2*H*-pyran-2-yl)anthracen-9(10*H*)-one	8015-61-0	O=C2C1=C(C([H])=C(C([H])=C1O[H])C([H])([H])O[H])[C@]([H])(C3=C2C(O[H])=C([H])C([H])=C3[H])[C@]4([H])O[C@@]([H])([C@@]([H])(O[H])[C@]([H])(O[H])[C@@]4([H])O[H])C([H])([H])O[H]	-0.59	-0.84	0.25	-0.79	0.20	-0.65	0.06	-0.62	0.03	T	Deng *et al.* 2007
**3**	Amoxicillin	(2*S*,5*R*,6*R*)-6-((R)-2-amino-2-(4-hydroxy-phenyl)acetamido)-3,3-dimethyl-7-oxo-4-thia-	26787-78-0	O=C2N1[C@]([H])([S]C([C@]1([H])C(=O)O[H])(C([H])([H])[H])C([H])([H])[H])[C@]2([H])N([H])	-0.92	-0.67	0.25	-0.78	0.14	-0.74	0.18	-0.67	0.25	T	Deng et al. 2007

		1-azabicyclo[3.2.0]heptane-2-carboxylic acid		C(=O)[C@]([H])(N([H])[H])C3=C([H])C([H])=C(O[H])C([H])=C3[H]										
**4**	Antipyrine	1,5-dimethyl-2-phenyl-1,2-dihydro-3*H*-pyrazol-3-one	60-80-0	O=C1N(N(/C(=C1/[H])C([H])([H])[H])C([H])([H])[H])C2=C([H])C([H])=C([H])C([H])=C2[H]	0.13	-0.12	0.25	-0.12	0.25	0.07	0.06	-0.03	0.16	T	Kim *et al.* 2006
**5**	Apigenin	5,7-dihydroxy-2-(4-hydroxyphenyl)-4*H*-chromen-4-one	520-36-5	O=C2C=3C(OC(/C1=C(\[H])C([H])=C(O[H])C([H])=C1[H])=C2[H])=C([H])C(O[H])=C([H])C=3O[H]	0.31	0.09	0.21	0.05	0.25	0.24	0.06	0.16	0.15	T	Fagerholm *et al.* 1997; Fagerholm *et al.* 1999; Kim *et al.* 2006; Lozoya-Agullo *et al.* 2015; Krondahl *et al.* 1997; Masaoka *et al.* 2006; Lindahl *et al.* 1999
**6**	Berberine	9,10-dimethoxy-5,6-dihydro-[1,3]dioxolo[4,5-*g*]isoquinolino[3,2	2086-83-1	[H]C3([H])[N+]4=C(C=2/C(=C(/[H])C=1OC([H])([H])OC=1C=2[H])C3([H])[H])C([H])	-0.66	-0.41	0.25	-0.89	0.22	-0.73	0.06	-0.82	0.16	T	Liu *et al.* 2002

		-*a*]iso-quino-lin-7-ium		=C5/C(=C4/[H])C(OC([H])([H])[H])=C(OC([H])([H])[H])C([H])=C5[H]										
**7**	Car-bamaz-epine	5*H*-dibenzo[*b*,*f*]aze pine-5-carbox-amide	298-46-4	O=C(N1C=3C(C([H])=C([H])C=2/C1=C(/[H])C([H])=C([H])C=2[H])=C([H])C([H])=C([H])C=3[H])N([H])[H]	0.08	-0.17	0.25	-0.17	0.25	0.02	0.06	-0.08	0.16	T	Patel et al. 2012; Fager-holm et al. 1997; Fager-holm et al. 1999; Kim et al. 2006; Lozoya-Agullo et al. 2015; Kron-dahl et al. 1997; Masaoka et al. 2006; Stef-fansen et al. 1999; Deng et al. 2007
**8**	Cephal exin	(6*R*,7*R*)-7-((*R*)-2-amino-2-phe-nyla-cetam-ido)-3-methyl-8-oxo-5-thia-1-azabi-cy-clo[4.2.0]oct-2-ene-2-	15686-71-2	O=C2N1C(=C(C([H])([H])[S][C@]1([H])[C@]2([H])N([H])C(=O)[C@]([H])(N([H])[H])C3=C([H])C([H])=C([H])C([H])=C3[H])C([H])([H]	-0.29	-0.59	0.30	-0.14	0.15	-0.35	0.06	-0.20	0.09	T	Stef-fansen *et al.* 1999

		carboxylic acid		)[H])C(=O)O[H]											
**9**	Chloramphenicol	2,2-dichloro-*N*-((1*R*,2*R*)-1,3-C13dihydroxy-1-(4-nitro+C18phenyl)propan-2-yl)acetamide	56-75-7	[Cl]C([Cl])([H])C(=O)N([H])[C@@]([H])([C@]([H])(O[H])C1=C([H])C([H])=C([N+]([O-])=O)C([H])=C1[H])C([H])([H])O[H]	0.04	-0.29	0.34	-0.21	0.25	-0.61	0.65	-0.12	0.16	T	Deng et al. 2007
**10**	Cimetidine	1-Cyano-2-methyl-3-(2-(((5-methyl-4-imidazolyl)methyl)thio)ethyl)guanidine	51481-61-9	N#CN([H])C(=NC([H])([H])[H])N([H])C([H])([H])C([H])([H])[S]C([H])([H])C=1/N=C(/[H])N([H])C=1C([H])([H])[H]	-1.08	-0.40	0.67	-0.83	0.25	-1.01	0.06	-0.92	0.16	T	Kim *et al.* 2006; Lozoya-Agullo *et al.* 2015
**11**	Codeine	(4*R*,4a*R*,7*S*,7a*R*,12b*S*)-9-methoxy-3-methyl-2,3,4,4a,7,7a-hexahydro-1*H*-4,12-methanobenzofuro[3,2-*e*]isoquinolin-7-ol	76-57-3	[H][C@]23C([H])=C([H])[C@]([H])(O[H])[C@]4([H])OC=1C5=C(C([H])=C([H])C=1OC([H])([H])[H])C([H])([H])[C@@]2([H])N(C([H])([H])C([H])(	-0.46	-0.71	0.25	-0.71	0.25	-0.52	0.06	-0.62	0.16	T	Kim *et al.* 2006; Lozoya-Agullo *et al.* 2015; Fang *et al.* 2016

				[H])[C@@]345)C([H])([H])[H]											
**12**	Colchicine	(*S*)-*N*-(1,2,3,10-tetramethoxy-9-oxo-5,6,7,9-tetrahydrobenzo[*a*]heptalen-7-yl)acetamide	64-86-8	O=C3C([H])=C2C(C=1/C(=C(/[H])C(OC([H])([H])[H])=C(OC([H])([H])[H])C=1OC([H])([H])[H])C([H])([H])C([H])([H])[C@]2([H])N([H])C(=O)C([H])([H])[H])=C([H])C([H])=C3OC([H])([H])[H]	-1.30	-1.05	0.25	-1.05	0.25	-1.23	0.06	-1.14	0.16	T	Chu *et al.* 2001; Steffansen *et al.* 1999
**13**	Creatinine	2-amino-1-methyl-1,5-dihydro-4*H*-imidazol-4-one	60-27-5	O=C1N=C(N(C1([H])[H])C([H])([H])[H])N([H])[H]	-1.68	-1.43	0.25	-1.43	0.25	-1.62	0.06	-1.52	0.16	T	Lozoya-Agullo *et al.* 2015
**14**	*D*-Asp(OBzl)–Ala	((*R*)-2-ammonio-4-(benzyloxy)-4-oxobutanoyl)-*L*-alaninate		O=C(OC([H])([H])C1=C([H])C([H])=C([H])C([H])=C1[H])C([H])([H])[C@]([H])(C(=O)N([H])[C@]([H])(C([O-])=O)C([H])([	0.11	-0.14	0.25	-0.14	0.25	0.05	0.06	-0.05	0.16	T	Kim *et al.* 2006a; Lozoya-Agullo *et al.* 2015; Dahan *et al.* 2009

				H])[H])[N+]([H])([H])[H]											
**15**	D-Glucose	(2*R*,3*R*,4*S*,5*S*,6*R*)-6-(hydroxymethyl)tetrahydro-2*H*-pyran-2,3,4,5-tetraol	2280-44-6	[H][C@]1(O[H])[C@]([H])(O[H])[C@@]([H])(O[H])[C@@]([H])(O[C@]1([H])C([H])([H])O[H])O[H]	-0.19	-0.45	0.25	0.05	0.25	-0.13	0.06	-0.12	0.08	T	Lozoya-Agullo *et al.* 2015
**16**	Enalaprilat	((*S*)-1-carboxy-3-phenylpropyl)-*L*-alanyl-*L*-proline	76420-72-9	O=C(N1[C@]([H])(C(=O)O[H])C([H])([H])C([H])([H])C1([H])[H])[C@@]([H])(N([H])[C@]([H])(C(=O)O[H])C([H])([H])C([H])([H])C2=C([H])C([H])=C([H])C([H])=C2[H])C([H])([H])[H]	-1.24	-0.18	1.06	-0.99	0.25	-1.08	0.16	-1.08	0.16	T	Lozoya-Agullo *et al.* 2015; Dahan *et al.* 2009
**17**	Fluvastatin	(3*R*,5*S*,*E*)-7-(3-(4-fluorophenyl)-1-isopropyl-1*H*-indol-2-yl)-3,5-dihydroxyhept-6-	93957-54-1	[F]/C3=C(\[H])C([H])=C(/C1=C(/N(C=2/C1=C(/[H])C([H])=C([H])C=2[H])C([H])(C([H])([H])[H])C([	-0.05	-0.30	0.25	-0.30	0.25	-0.11	0.06	-0.20	0.16	T	Fagerholm *et al.* 1999

		enoic acid		H])([H])[H])C([H])=C([H])[C@@]([H])(O[H])C([H])([H])[C@@]([H])(O[H])C([H])([H])C(=O)O[H])C([H])=C3[H]										
**18**	Furosemide	4-chloro-2-((furan-2-ylmethyl)amino)-5-sulfamoyl benzoic acid	54-31-9	[Cl]/C2=C(/C([H])=C(C(N([H])C([H])([H])C=1OC([H])=C([H])C=1[H])=C2[H])C(=O)O[H])[S](=O)(=O)N([H])[H]	-0.84	-0.45	0.39	-0.27	0.57	0.28	1.11	-0.34	0.50	T	Deng et al. 2007
**19**	Gabapentin	2-(1-(aminomethyl)cyclohexyl)acetic acid	60142-96-3	O=C(O[H])C([H])([H])C1(C([H])([H])C([H])([H])C([H])([H])C([H])([H])C1([H])[H])C([H])([H])N([H])[H]	-1.25	-0.57	0.68	-0.69	0.56	-1.18	0.06	-1.41	0.16	T	Steffansen *et al.* 1999
**20**	Genistein	5,7-dihydroxy-3-(4-hydroxyphenyl)-4*H*-chromen-4-one	446-72-0	O=C1C=3C(OC([H])=C1/C2=C(\[H])C([H])=C(O[H])C([H])=C2[H])=C([H])C(O[H])=C([H])	0.21	-0.04	0.25	-0.04	0.25	0.15	0.06	0.06	0.15	T	Fagerholm *et al.* 1996; Fagerholm *et al.* 1997; Krondahl *et al.* 1997; Lindahl

				C=3O[H]											*et al.* 1999; Lozoya-Agullo *et al.* 2015; Kim *et al.* 2006; Gullo *et al.* 2015; Dahan *et al.* 2009
**21**	Griseofulvin	(2*S*,6'*R*)-7-chloro-2',4,6-trimethoxy-6'-methyl-3*H*-spiro[benzofuran-2,1'-cyclohexan]-2'-ene-3,4'-dione	126-07-8	[Cl]C=2C=1O[C@]3(C(=O)C=1C(OC([H])([H])[H])=C([H])C=2OC([H])([H])[H])C(OC([H])([H])[H])=C([H])C(=O)C([H])([H])[C@@]3([H])C([H])([H])[H]	-0.04	-0.71	0.67	-0.29	0.25	-0.10	0.06	0.09	0.14	T	Lozoya-Agullo *et al.* 2015
**22**	Hydrochlorothiazide	6-chloro-3,4-dihydro-2*H*-benzo[*e*][1,2,4]thiadiazine-7-sulfonamide 1,1-dioxide	58-93-5	[Cl]/C2=C(/C([H])=C1C(N([H])C([H])([H])N([H])[S]1(=O)=O)=C2[H])[S](=O)(=O)N([H])[H]	-3.00	-2.18	0.82	-2.75	0.25	-2.94	0.06	-3.16	0.16	T	Kim *et al.* 2006
**23**	Ibuprofen	(*R*)-2-(4-isobutylphenyl)propanoic acid	15687-27-1	O=C(O[H])[C@@]([H])(C1=C([H])C([H])=C(C([H])=C1[H])C([H])([H]	0.34	0.23	0.12	0.27	0.07	0.28	0.06	0.19	0.15	T	Fagerholm *et al.* 1997; Lindahl *et al.* 1998; Lindahl *et al.* 1999

			)C([H])(C([H])([H])[H])C([H])([H])[H])C([H])([H])[H]											
**24**	II	*tert*-butyl ((2*S*,3*R*,4*S*)-1-cyclohexyl-3,4-dihydroxy-6-methylheptan-2-yl)carbamate	O=C(OC(C([H])([H])[H])(C([H])([H])[H])C([H])([H])[H])N([H])[C@@]([H])(C([H])([H])[C@]1([H])C([H])([H])C([H])([H])C([H])([H])C([H])([H])C1([H])[H])[C@@]([H])(O[H])[C@@]([H])(O[H])C([H])([H])C([H])(C([H])([H])[H])C([H])([H])[H]	0.17	-0.04	0.21	0.19	0.02	0.11	0.06	0.04	0.13	T	Fagerholm *et al.* 1996; Kim *et al.* 2006; Lozoya-Agullo *et al.* 2015; Fang *et al.* 2016
**25**		2-amino-N-((2*S*,3*R*,4*S*)-1-cyclohexyl-3,4-dihydroxy-6-methylheptan-2-yl)acetamide	O=C(N([H])[C@@]([H])(C([H])([H])[C@]1([H])C([H])([H])C([H])([H])C([H])([H])C([H])([H])C1([H])[H])[C@@]([H])(O[	-0.70	-0.74	0.04	-0.94	0.25	-0.76	0.06	-0.86	0.16	T	Stewart *et al.* 1995

				H])[C@@]([H])(O[H])C([H])([H])C([H])(C([H])([H])[H])C([H])([H])[H])C([H])([H])N([H])[H]										
**26**	Isofraxidin	7-hydroxy-6,8-dimethoxy-2*H*-chromen-2-one	486-21-5	O=C2OC=1/C(=C(/[H])C(OC([H])([H])[H])=C(O[H])C=1OC([H])([H])[H])C([H])=C2[H]	-0.40	-0.65	0.25	-0.65	0.25	-0.47	0.06	-0.56	0.15	T	Liu *et al.* 2002
**27**		(*S*)-*N*-((2*S*,3*R*,4*S*)-1-cyclohexyl-3,4-dihydroxy-6-methylheptan-2-yl)-3-phenyl-2-(thiomorpholine-4-sulfonamido)propanamide		O=C(N([H])[C@@]([H])(C([H])([H])[C@@]1([H])C([H])([H])C([H])([H])C([H])([H])C([H])([H])C1([H])[H])[C@@]([H])(O[H])[C@@]([H])(O[H])C([H])([H])C([H])(C([H])([H])[H])C([H])([H])[H])[C@@]([H])(N([H])[S](=O)(=O)N2C([H])([H]	-0.44	-0.46	0.02	-0.49	0.04	-0.51	0.06	-0.28	0.16	T	Jain *et al.* 2008

				)C([H])([H])[S]C([H])([H])C2([H])[H])C([H])([H])C=3C([H])=C([H])C([H])=C([H])C=3[H]											
**28**	Labetalol	2-hydroxy-5-((*S*)-1-hydroxy-2-(((*S*)-4-phenylbutan-2-yl)amino)ethyl)benzamide	36894-69-6	O=C(N([H])[H])C=1C([H])=C(C([H])=C([H])C=1O[H])[C@]([H])(O[H])C([H])([H])N([H])[C@]([H])(C([H])([H])C([H])([H])C2=C([H])C([H])=C([H])C([H])=C2[H])C([H])([H])[H]	-0.49	-0.74	0.25	-0.24	0.25	-0.48	0.01	-0.33	0.16	T	Masaoka *et al.* 2006
**29**	Lamivudine	4-amino-1-((2*R*,5*S*)-2-(hydroxymethyl)-1,3-oxathiolan-5-yl)pyrimidin-2(1*H*)-one	134678-17-4	O=C1/N=C(/N([H])[H])C([H])=C([H])N1[C@@]2([H])O[C@]([H])([S]C2([H])[H])C([H])([H])O[H]	-0.48	-0.73	0.25	-0.73	0.25	-0.50	0.02	-0.67	0.18	T	Kim *et al.* 2006
**30**	L-Dopa	(*S*)-2-amino-3-(3,4-dihydroxyphenyl)	59-92-7	O=C(O[H])[C@@]([H])(N([H])[H])C([H])(	0.30	-0.58	0.88	-0.20	0.50	0.24	0.06	0.14	0.16	T	Stewart *et al.* 1995

		propanoic acid		[H])C1=C([H])C(O[H])=C(O[H])C([H])=C1[H]											
**31**	L-Phenylalanine	(*S*)-2-Amino-3-phenylpropionic acid	63-91-2	O=C(O[H])[C@@]([H])(N([H])[H])C([H])([H])C1=C([H])C([H])=C([H])C([H])=C1[H]	0.25	-0.07	0.32	-0.12	0.37	-0.34	0.60	0.09	0.16	T	Lozoya-Agullo *et al.* 2015
**32**	Methyldopa	(*S*)-2-amino-3-(3,4-dihydroxyphenyl)-2-methylpropanoic acid	555-30-6	O=C(O[H])[C@@](N([H])[H])(C([H])([H])C1=C([H])C(O[H])=C(O[H])C([H])=C1[H])C([H])([H])[H]	-1.80	-0.62	1.18	-0.88	0.91	-0.38	1.42	-1.49	0.31	T	Stewart *et al.* 1995
**33**	Metoprolol	(*R*)-1-(isopropylamino)-3-(4-(2-methoxyethyl)phenoxy)propan-2-ol	51384-51-1	[H][C@@](O[H])(C([H])([H])N([H])C([H])(C([H])([H])[H])C([H])([H])[H])C([H])([H])O/C1=C(\[H])C([H])=C(C([H])=C1[H])C([H])([H])C([H])([H])OC([H])([H])[H]	-0.39	-0.14	0.25	-0.44	0.05	-0.33	0.06	-0.17	0.22	T	Stewart *et al.* 1995
**34**	Naproxen	(*S*)-2-(6-	22204-53-1	O=C(O[H])[C@]([H]	0.15	-0.10	0.25	-0.10	0.25	0.21	0.06	-0.01	0.16	T	Deng et al. 2007

		methoxynaphthalen-2-yl)propanoic acid		)(C1=C([H])C2=C(C([H])=C1[H])C([H])=C(OC([H])([H])[H])C([H])=C2[H])C([H])([H])[H]										
**35**	Naringenin	(*S*)-5,7-dihydroxy-2-(4-hydroxyphenyl)chroman-4-one	480-41-1	O=C2C=3C(O[C@]([H])(C1=C([H])C([H])=C(O[H])C([H])=C1[H])C2([H])[H])=C([H])C(O[H])=C([H])C=3O[H]	-0.34	-0.13	0.21	-0.07	0.27	-0.17	0.17	0.01	0.35	T	Stewart *et al.* 1995
**36**	Olmesartan	1-((2'-(2*H*-tetrazol-5-yl)-[1,1'-biphenyl]-4-yl)methyl)-4-(2-hydroxypropan-2-yl)-2-propyl-1*H*-imidazole-5-carboxylic acid	144689-24-7	O=C(O[H])C=1N(C(=NC=1C(O[H])(C([H])([H])[H])C([H])([H])[H])C([H])([H])C([H])([H])C([H])([H])[H])C([H])([H])C2=C([H])C([H])=C(C([H])=C2[H])C=3/C(=C(/[H])C([H])=C([H])C=3[H])C4=NN([H])N=N4	-1.70	-1.45	0.25	-1.45	0.25	-1.64	0.06	-1.55	0.14	T	Fagerholm *et al.* 1996; Kim *et al.* 2006
**37**	Phenol red	3,3-*bis*(4-	143-74-8	O=[S]1(=O)O	-0.12	-0.37	0.25	-0.37	0.25	-0.18	0.06	-0.28	0.16	T	Incecayir *et al.*

		hydroxy-phenyl)-3*H*-benzo[*c*][1,2]oxathiole 1,1-dioxide		C(C=2/C1=C(/[H])C([H])=C([H])C=2[H])(C3=C([H])C([H])=C(O[H])C([H])=C3[H])C4=C([H])C([H])=C(O[H])C([H])=C4[H]											2013; Lozoya-Agullo *et al.* 2015
**38**	Pindolol	(*S*)-1-((1*H*-indol-4-yl)oxy)-3-(isopropylamino)propan-2-ol	13523-86-9	[H][C@](O[H])(C([H])([H])N([H])C([H])(C([H])([H])[H])C([H])([H])[H])C([H])([H])OC=2C1=C(N([H])C([H])=C1[H])C([H])=C([H])C=2[H]	-0.53	-0.76	0.23	-0.62	0.09	-0.47	0.06	-0.43	0.10	T	Patel *et al.* 2012
**39**	Piroxicam	4-hydroxy-2-methyl-*N*-(pyridin-2-yl)-2*H*-benzo[*e*][1,2]thiazine-3-carboxamide 1,1-dioxide	36322-90-4	O=[S]1(=O)N(/C(=C(/O[H])C=2/C1=C(/[H])C([H])=C([H])C=2[H])C(=O)N([H])C=3/N=C(/[H])C([H])=C([H])C=3[H])C([H])([H])[H]	0.42	-0.57	0.99	-0.10	0.52	0.21	0.21	0.37	0.05	T	Fagerholm *et al.* 1997
**40**	Propranolol	(*S*)-1-(iso-	525-66-6	[H][C@](O[H])(C([	-0.19	0.06	0.25	-0.44	0.25	-0.12	0.06	-0.35	0.16	T	Krondahl *et*

		propylamino)-3-(naphthalen-1-yloxy)propan-2-ol		H])([H])N([H])C([H])(C([H])([H])[H])C([H])([H])[H])C([H])([H])OC=1C=2C(C([H])=C([H])C=1[H])=C([H])C([H])=C([H])C=2[H]											*al.* 1997
**41**	Ranitidine	(*E*)-*N*-(2-(((5-((dimethylamino)methyl)furan-2-yl)methyl)thio)ethyl)-*N*'-methyl-2-nitroethene-1,1-diamine	66357-35-5	[O-][N+](=O)C([H])=C(N([H])C([H])([H])C([H])([H])[S]C([H])([H])C=1O/C(=C(/[H])C=1[H])C([H])([H])N(C([H])([H])[H])C([H])([H])[H])N([H])C([H])([H])[H]	-1.14	-1.23	0.09	-0.89	0.25	-1.07	0.06	-0.98	0.16	T	Krondahl *et al.* 1997
**42**	Rhein	4,5-dihydroxy-9,10-dioxo-9,10-dihydroanthracene-2-carboxylic acid	478-43-3	O=C1C3=C(C(=O)C2=C1C(O[H])=C([H])C([H])=C2[H])C([H])=C(C([H])=C3O[H])C(=O)O[H]	-0.18	-0.35	0.17	-0.25	0.07	-0.28	0.11	-0.07	0.10	T	Stewart *et al.* 1995
**43**	Tanshinone I	1,6-dimethylphe	54693-68-4	O=C2C3=C(C=1OC([H])=C(	-0.07	0.17	0.24	-0.23	0.16	0.00	0.06	-0.08	0.01	T	Kim *et al.* 2006; Stewart

		nanthro[1,2-*b*]furan-10,11-dione		C=1C2=O)C([H])([H])[H])C([H])=C([H])C=4/C3=C(/[H])C([H])=C([H])C=4C([H])([H])[H]											*et al.* 1995; Masaoka *et al.* 2006
**44**	Tanshinone IIA	1,6,6-trimethyl-6,7,8,9-tetrahydrophenanthro[1,2-*b*]furan-10,11-dione	568-72-9	O=C3C=1C4=C(C([H])=C([H])C=1C=2OC([H])=C(C=2C3=O)C([H])([H])[H])C(C([H])([H])C([H])([H])C4([H])[H])(C([H])([H])[H])C([H])([H])[H]	-0.19	0.06	0.25	-0.16	0.03	-0.12	0.06	0.04	0.22	T	Kim *et al.* 2006
**45**	TAPP	(*S*)-2-amino-*N*-((*R*)-1-(((*S*)-1-(((*S*)-1-amino-1-oxo-3-phenylpropan-2-yl)amino)-1-oxo-3-phenylpropan-2-yl)amino)-1-oxopropan-2-yl)-3-(4-hydroxyphenyl)		O=C(N([H])[C@@]([H])(C(=O)N([H])[C@]([H])(C(=O)N([H])[C@]([H])(C(=O)N([H])[H])C([H])([H])C=1C([H])=C([H])C([H])=C([H])C=1[H])C([H])([H])C=2C([H])=C([H])C([H])=C(	-0.17	-0.42	0.25	-0.42	0.25	-0.23	0.06	-0.33	0.16	T	Fagerholm *et al.* 1997; Incecayir *et al.* 2013; Kim *et al.* 2006; Lozoya-Agullo *et al.* 2015; Ozawa *et al.* 2014; Dahan *et al.* 2009; Krondahl *et al.* 1997; Masaok

		propanamide		[H])C=2[H])C([H])([H])[H])[C@@]([H])(N([H])[H])C([H])([H])C=3C([H])=C([H])C(O[H])=C([H])C=3[H]											a *et al.* 2006
**46**		5-fluoro-3-(2-methylbenzoyl)pyrimidine-2,4(1*H*,3*H*)-dione		[F]C=2C(=O)N(C(=O)C=1/C(=C(/[H])C([H])=C([H])C=1[H])C([H])([H])[H])C(=O)N([H])C=2[H]	-1.18	-0.45	0.74	-0.80	0.39	-0.41	0.78	-1.14	0.05	T	Ozawa *et al.* 2014
**47**	Theophylline	1,3-dimethyl-3,7-dihydro-1*H*-purine-2,6-dione	58-55-9	O=C2N(C(=O)N(C=1/N=C(/[H])N([H])C=12)C([H])([H])[H])C([H])([H])[H]	-0.17	-0.40	0.24	-0.03	0.14	-0.10	0.06	-0.01	0.16	T	Fagerholm *et al.* 1997; Kim *et al.* 2006; Masaoka *et al.* 2006
**48**	Urea	urea	57-13-6	O=C(N([H])[H])N([H])[H]	-0.15	-0.35	0.19	-0.25	0.09	-0.14	0.01	-0.11	0.05	T	Deng *et al.* 2007
**49**		*N*-((2*S*,3*R*,4*S*)-1-cyclohexyl-3,4-dihydroxy-6-methylheptan-2-yl)-6-phenylhexanamide		O=C(N([H])[C@@]([H])(C([H])([H])[C@]1([H])C([H])([H])C([H])([H])C([H])([H])C([H])([H])C1([H])[H])[C@@]([	0.18	0.10	0.08	-0.07	0.25	0.12	0.06	0.02	0.16	T	Kang *et al.* 2012

				H])(O[H])[C@@]([H])(O[H])C([H])([H])C([H])(C([H])([H])[H])C([H])([H])[H])C([H])([H])C([H])([H])C([H])([H])C([H])([H])C([H])([H])C=2C([H])=C([H])C([H])=C([H])C=2[H]											
**50**	Valsartan	*N*-((2'-(2H-tetrazol-5-yl)-[1,1'-biphenyl]-4-yl)methyl)-*N*-pentanoyl-*L*-valine	137862-53-4	O=C(N([C@]([H])(C(=O)O[H])C([H])(C([H])([H])[H])C([H])([H])[H])C([H])([H])C1=C([H])C([H])=C(C([H])=C1[H])C=2/C(=C(/[H])C([H])=C([H])C=2[H])C3=NN([H])N=N3)C([H])([H])C([H])([H])C([H])([H])C([H])([H])[H]	-1.22	-0.97	0.25	-0.97	0.25	-1.28	0.06	-1.38	0.16	T	Kang *et al.* 2012
**51**	Verapamil	(*R*)-5-((3,4-	52-53-9	N#C[C@@](/	-0.19	-0.32	0.14	-0.44	0.25	-0.25	0.06	-0.35	0.16	T	Shashikanth *et*

	di-methoxyphenethyl)(*m*+C54ethyl)amino)-2-(3,4-di-methoxyphenyl)-2-iso-propylpentane-nitrile	C1=C(\[H])C(OC([H])([H])[H])=C(OC([H])([H])[H])C([H])=C1[H])(C([H])(C([H])([H])[H])C([H])([H])[H])C([H])([H])C([H])([H])C([H])([H])N(C([H])([H])C([H])([H])C2=C([H])C(OC([H])([H])[H])=C(OC([H])([H])[H])C([H])=C2[H])C([H])([H])[H]										*al.* 2013a
**52**	*N*2-(tert-butoxycar-bonyl)-*N*4-((2*S*,3*R*,4*S*)-1-cyclo-hexyl-3,4-di-hy-droxy-6-methylheptan-2-yl)-*L*-aspara-gine	O=C(N([H])[C@@]([H])(C([H])([H])[C@]1([H])C([H])([H])C([H])([H])C([H])([H])C([H])([H])C1([H])[H])[C@@]([H])(O[H])[C@@]([H])(O[H])C([H])([H])C([H])(C([H])([H])[H	-1.01	-0.76	0.25	-0.76	0.25	-0.94	0.06	-0.85	0.15	T	Fang *et al.* 16a

				])C([H])([H])[H])C([H])([H])[C@]([H])(N([H])C(=O)OC(C([H])([H])[H])(C([H])([H])[H])C([H])([H])[H])C(=O)O[H]										
**53**	VII	*N*-((2*S*,3*R*,4*S*)-1-cyclohexyl-3,4-dihydroxy-6-methylheptan-2-yl)cinnamamide		O=C(N([H])[C@@]([H])(C([H])([H])[C@]1([H])C([H])([H])C([H])([H])C([H])([H])C([H])([H])C1([H])[H])[C@@]([H])(O[H])[C@@]([H])(O[H])C([H])([H])C([H])(C([H])([H])[H])C([H])([H])[H])C([H])=C([H])C=2C([H])=C([H])C([H])=C([H])C=2[H]	0.14	0.14	0.00	0.39	0.25	0.20	0.06	-0.02	0.16	T	Deng et al. 2007
**54**	Aloe-emodin	1,8-dihydroxy-3-(hydroxymethyl)a	481-72-1	O=C1C3=C(C(=O)C2=C1C(O[H])=C([H])C([H])C([H])	-0.16	0.07	0.23	-0.03	0.13	-0.19	0.03	0.07	0.23	t	Kim *et al.* 2006

		nthracene-9,10-dione		=C2[H])C([H])=C(C([H])=C3O[H])C([H])([H])O[H]										
**55**	Atenolol	(*R*)-2-(4-(2-hydroxy-3-(isopropylamino)propoxy)phenyl)acetamide	29122-68-7	O=C(N([H])[H])C([H])([H])C1=C([H])C([H])=C(OC([H])([H])[C@]([H])(O[H])C([H])([H])N([H])C([H])(C([H])([H])[H])C([H])([H])[H])C([H])=C1[H]	-1.02	-0.99	0.03	-1.65	0.63	-1.65	0.64	-1.19	0.17	t	Kim *et al.* 2006
**56**	Benzylalcochol	phenylmethanol	100-51-6	[H]C([H])(O[H])C1=C([H])C([H])=C([H])C([H])=C1[H]	0.19	0.40	0.21	-0.42	0.61	0.00	0.19	-0.22	0.41	t	Stewart *et al.* 1995
**57**	Caffeine	1,3,7-trimethyl-3,7-dihydro-1*H*-purine-2,6-dione	58-08-2	O=C1N(C(=O)N(C=2/N=C(/[H])N(C1=2)C([H])([H])[H])C([H])([H])[H])C([H])([H])[H]	0.06	-0.38	0.44	0.12	0.06	0.01	0.05	0.00	0.05	t	Kim *et al.* 2006; Dahan *et al.* 2009; Nagare *et al.* 2010; Patel *et al.* 2012
**58**	Cryptotanshinone	(*R*)-1,6,6-trimethyl-1,2,6,7,8,9-hexahydrophenanthro[1,2	35825-57-1	O=C3C=1C4=C(C([H])=C([H])C=1C=2OC([H])([H])[C@@]([H])(C=2C3=O	-0.15	0.04	0.19	-0.27	0.12	-0.21	0.06	0.03	0.18	t	Kim *et al.* 2006

		-*b*]furan-10,11-dione		)C([H])([H])[H])C(C([H])([H])C([H])([H])C4([H])[H])(C([H])([H])[H])C([H])([H])[H]											
**59**		(2*S*,3*R*,4*S*)-2-amino-1-cyclohexyl-6-methylheptane-3,4-diol		[H]C1([H])C([H])([H])C([H])([H])[C@]([H])(C([H])([H])C1([H])[H])C([H])([H])[C@]([H])(N([H])[H])[C@@]([H])(O[H])[C@@]([H])(O[H])C([H])([H])C([H])(C([H])([H])[H])C([H])([H])[H]	-0.35	0.01	0.35	-0.73	0.38	-0.58	0.24	-0.31	0.04	t	Deng et al. 2007
**60**	Ketoprofen	(*R*)-2-(3-benzoylphenyl)propanoic acid	22071-15-4	O=C(C=1C([H])=C(C([H])=C([H])C=1[H])[C@]([H])(C(=O)O[H])C([H])([H])[H])C2=C([H])C([H])=C([H])C([H])=C2[H]	0.30	-0.15	0.45	0.24	0.05	0.47	0.17	0.70	0.40	t	Jain *et al.* 2008
**61**	Mannitol	(2*R*,3*R*,4*R*,5*R*)-hexane-1,2,3,4,	69-65-8	[H][C@](O[H])([C@]([H]	-1.18	-0.19	0.99	-0.90	0.27	-0.78	0.40	-0.71	0.47	t	Deng et al. 2007

		5,6-hexaol		)(O[H])[C@]([H])(O[H])C([H])([H])O[H])[C@]([H])(O[H])C([H])([H])O[H]										
**62**	Minoxidil	6-amino-2-imino-4-(piperidin-1-yl)pyrimidin-1(2*H*)-ol	38304-91-5	[H]C2([H])C([H])([H])C([H])([H])N(/C1=N/C(=N\[H])N(O[H])C(N([H])[H])=C1[H])C([H])([H])C2([H])[H]	-0.66	-0.54	0.11	-0.49	0.16	-0.60	0.05	-0.34	0.32	t	Deng *et al.* 2007
**63**	Olmesartan medoxomil	(5-methyl-2-oxo-1,3-dioxol-4-yl)methyl 1-((2'-(2*H*-tetrazol-5-yl)-[1,1'-biphenyl]-4-yl)methyl)-4-(2-hydroxypropan-2-yl)-2-propyl-1*H*-imidazole-5-carboxylate	144689-63-4	O=C(OC([H])([H])C=1OC(=O)OC=1C([H])([H])[H])C=2N(C(=NC=2C(O[H])(C([H])([H])[H])C([H])([H])[H])C([H])([H])C([H])([H])C([H])([H])[H])C([H])([H])C3=C([H])C([H])=C(C([H])=C3[H])C=4/C(=C(/[H])C([H])=C([H])C=4[H])C5=	-0.97	-0.51	0.47	-1.19	0.22	-1.01	0.04	-1.10	0.13	t	Krondahl *et al.* 1997

				NN([H])N=N5											
**64**	Physcion	1,8-dihydroxy-3-methoxy-6-methylanthracene-9,10-dione	521-61-9	O=C1C3=C(C(=O)C2=C1C(O[H])=C([H])C(OC([H])([H])[H])=C2[H])C([H])=C(C([H])=C3O[H])C([H])([H])[H]	-0.19	0.42	0.60	-0.14	0.05	0.31	0.50	-0.03	0.16	t	Fagerholm *et al.* 1996
**65**	Prednisolone	(8*S*,9*S*,10*R*,11*S*,13*S*,14*S*,17*R*)-11,17-dihydroxy-17-(2-hydroxyacetyl)-10,13-dimethyl-6,7,8,9,10,11,12,13,14,15,16,17-dodecahydro-3*H*-cyclopenta[*a*]phenanthren-3-one	50-24-8	O=C([C@]4(O[H])[C@@]1([C@]([H])([C@@]3([H])[C@]([H])([C@@]([H])(O[H])C1([H])[H])[C@@]2(/C(=C(/[H])C(=O)C([H])=C2[H])C([H])([H])C3([H])[H])C([H])([H])[H])C([H])([H])C4([H])[H])C([H])([H])[H])C([H])([H])O[H]	-0.52	-0.39	0.13	0.16	0.68	-0.17	0.35	-0.36	0.16	t	Liu *et al.* 2010
**66**	Terbutaline	(*R*)-5-(2-(*tert*-butylamino)-1-hydroxyethyl)b	23031-25-6	[H]N(C(C([H])([H])[H])(C([H])([H])[H])C([H])([H])[H])C(	-1.35	-0.53	0.82	-1.37	0.02	-1.00	0.34	-1.21	0.14	t	Kim *et al.* 2006

		enzene-1,3-diol		[H])([H])[C@]([H])(O[H])C1=C([H])C(O[H])=C([H])C(O[H])=C1[H]											
**67**	Digoxin	4-[(3*S*,5*R*,8*R*,9*S*,10*S*,12*R*,13*S*,14*S*)-3-[(2*S*,4*S*,5*R*,6*R*)-5-[(2*S*,4*S*,5*R*,6*R*)-5-[(2*S*,4*S*,5*R*,6*R*)-4,5-dihydroxy-6-methyloxan-2-yl]oxy-4-hydroxy-6-methyloxan-2-yl]oxy-4-hydroxy-6-methyloxan-2-yl]oxy-12,14-dihydroxy-10,13-dimethyl-1,2,3,4,5,6,7,8,9,11,12,15,16,17-tetradecahydrocyclopenta[*a*]phe-	20830-75-5	O=C1OC([H])([H])C(=C1[H])[C@]8([H])[C@]7([C@]([H])(O[H])C([H])([H])[C@@]6([H])[C@@]([H])(C([H])([H])C([H])([H])[C@]5([H])C([H])([H])[C@@]([H])(O[C@]4([H])O[C@@]([H])([C@@]([H])(O[C@]3([H])O[C@@]([H])([C@@]([H])(O[C@]2([H])O[C@@]([H])([C@@]([H])(O[H])[C@@]([H])(O[H])C2([H])[H])C([H])([H])[H])[C@@]([H])(	-0.52	-0.56	0.04	-0.89	0.37	-0.74	0.22	-0.84	0.31	o	Fagerholm *et al.* 1999; Masaoka *et al.* 2006

		nanthren-17-yl]-5H-furan-2-one	O[H])C3([H])[H])C([H])([H])[H])[C@@]([H])(O[H])C4([H])[H])C([H])([H])[H])C([H])([H])C([H])([H])[C@]56C([H])([H])[H])[C@@]7(O[H])C([H])([H])C8([H])[H])C([H])([H])[H]											
**68**	Gly-Val-SQV	(2*R*,3*S*)-3-((*S*)-4-amino-4-oxo-2-(quinoline-2-carboxamido)butanamido)-1-((3*S*,4a*S*,8a*R*)-3-(*tert*-butylcarbamoyl)octahydroisoquinolin-2(1*H*)-yl)-4-phenylbutan-2-yl glycyl-L-valinate	O=C(N([H])C(C([H])([H])[H])(C([H])([H])[H])C([H])([H])[H])[C@@]1([H])N(C([H])([H])[C@@]2([H])[C@]([H])(C1([H])[H])C([H])([H])C([H])([H])C([H])([H])C2([H])[H])C([H])([H])[C@@]([H])(OC(=O)[C@@]([H])(N([H])C(=O)C([H])([H])N([H])	-0.38	-0.23	0.15	-0.71	0.34	-0.64	0.27	-0.44	0.07	o	Stewart *et al.* 1995

			[H])C([H])(C([H])([H])[H])C([H])([H])[H])[C@@]([H])(N([H])C(=O)[C@@]([H])(N([H])C(=O)C3=NC=4C(C([H])=C3[H])=C([H])C([H])=C([H])C=4[H])C([H])([H])C(=O)N([H])[H])C([H])([H])C=5C([H])=C([H])C([H])=C([H])C=5[H]											
**69**	LEF537	(*R*)-*N*-((*S*)-1-(((*S*)-1-amino-1-oxopentan-2-yl)amino)-1-oxo-3-phenylpropan-2-yl)-2-((*S*)-2-amino-3-(4-hydroxyphenyl)propanamido)-5-guanidinopentanamide	O=C(N([H])[C@@]([H])(C(=O)N([H])[C@]([H])(C(=O)N([H])[C@]([H])(C(=O)N([H])[H])C([H])([H])C([H])([H])C([H])([H])[H])C([H])([H])C=1C([H])=C([H])C([H])=C([H])C=1[H])C([H])([H]	-0.11	0.03	0.14	-0.75	0.64	-0.62	0.52	-0.29	0.19	o	Lozoya-Agullo *et al.* 15a

			])C([H])([H])C([H])([H])N([H])C(=N[H])N([H])[H])[C@@]([H])(N([H])[H])C([H])([H])C=2C([H])=C([H])C(O[H])=C([H])C=2[H]										
**70**	LEF553	(*R*)-*N*-((*S*)-1-(((*S*)-1-amino-1-oxo-3-phenylpropan-2-yl)amino)-1-oxo-3-phenylpropan-2-yl)-2-((*S*)-2-amino-3-(4-hydroxyphenyl)propanamido)-5-guanidinopentanamide	O=C(N([H])[C@@]([H])(C(=O)N([H])[C@]([H])(C(=O)N([H])[C@]([H])(C(=O)N([H])[H])C([H])([H])C=1C([H])=C([H])C([H])=C([H])C=1[H])C([H])([H])C=2C([H])=C([H])C([H])=C([H])C=2[H])C([H])([H])C([H])([H])C([H])([H])N([H])C(=N[H])N([H])[H])[C@@]([H])(N([H])[H])C([H])([H]	-0.37	0.07	0.44	-0.71	0.35	-0.62	0.25	-0.18	0.19	o	Jain *et al.* 2008

				])C=3C([H])=C([H])C(O[H])=C([H])C=3[H]											
**71**	Paclitaxel	(2a*R*,4*S*,4a*S*,6*S*,7*S*,8*R*,9*S*,11*S*,12*S*,12a*R*,12b*S*)-9-(((2*R*,3*S*)-3-benzamido-2-hydroxy-3-phenylpropanoyl)oxy)-12-(benzoyloxy)-4,11-dihydroxy-4a,8,13,13-tetramethyl-5-oxododecahydro-1*H*-7,11-methanocyclodeca[3,4]benzo[1,2-*b*]oxete-6,12b(2a*H*)-diyl diacetate	33069-62-4	O=C4[C@]2([C@@]([H])([C@@]1(OC(=O)C([H])([H])[H])[C@]([H])(OC1([H])[H])C([H])([H])[C@]2([H])O[H])[C@]([H])(OC(=O)C3=C([H])C([H])=C([H])C([H])=C3[H])[C@@]7(O[H])C([C@@]([H])([C@]4([H])OC(=O)C([H])([H])[H])[C@]([H])([C@@]([H])(OC(=O)[C@]([H])(O[H])[C@@]([H])(N([H])C(=O)C=5C([H])=C([H])C([H])=C([H])C=5[H])C6=C([H])C([H])=C	-0.64	-0.20	0.44	-0.91	0.27	-0.66	0.02	-0.82	0.18	o	Kim *et al.* 2006

			([H])C([H])=C6[H])C7([H])[H])C([H])([H])[H])(C([H])([H])[H])C([H])([H])[H])C([H])([H])[H]										
**72**	Saquinavir	(*S*)-*N*1-((2*S*,3*R*)-4-((3*S*,4a*S*,8a*R*)-3-(*tert*-butylcarbamoyl)octahydroisoquinolin-2(1*H*)-yl)-3-hydroxy-1-phenylbutan-2-yl)-2-(quinoline-2-carboxamido)succinamide	127779-20-8	O=C(N([H])C(C([H])([H])[H])(C([H])([H])[H])C([H])([H])[H])[C@@]1([H])N(C([H])([H])[C@@]2([H])[C@]([H])(C1([H])[H])C([H])([H])C([H])([H])C([H])([H])C2([H])[H])C([H])([H])[C@@]([H])(O[H])[C@@]([H])(N([H])C(=O)[C@@]([H])(N([H])C(=O)C3=NC=4C(C([H])=C3[H])=C([H])C([H])=C([H])C=4[H])C([H])([H])C	-0.64	-0.35	0.28	-0.62	0.02	-0.46	0.18	-0.50	0.14	o	Stewart *et al.* 1995

			(=O)N([H])[H])C([H])([H])C=5C([H])=C([H])C([H])=C([H])C=5[H]											
**73**	Val-Val-SQV	(2*R*,3*S*)-3-((S)-4-amino-4-oxo-2-(quinoline-2-carboxamido)butanamido)-1-((3*S*,4a*S*,8a*R*)-3-(*tert*-butylcarbamoyl)octahydroisoquinolin-2(1*H*)-yl)-4-phenylbutan-2-yl *L*-valyl-*L*-valinate	O=C(N([H])C(C([H])([H])[H])(C([H])([H])[H])C([H])([H])[H])[C@@]1([H])N(C([H])([H])[C@@]2([H])[C@]([H])(C1([H])[H])C([H])([H])C([H])([H])C([H])([H])C2([H])[H])C([H])([H])[C@@]([H])(OC(=O)[C@@]([H])(N([H])C(=O)[C@@]([H])(N([H])[H])C([H])(C([H])([H])[H])C([H])([H])[H])C([H])(C([H])([H])[H])C([H])([H])[H])[C@@]([H])(N([H])C(=	0.11	-0.10	0.21	-0.46	0.57	-0.35	0.46	-0.15	0.26	o	Stewart *et al.* 1995

		O)[C@@]([H])(N([H])C(=O)C3=NC=4C(C([H])=C3[H])=C([H])C([H])=C([H])C=4[H])C([H])([H])C(=O)N([H])[H])C([H])([H])C=5C([H])=C([H])C([H])=C([H])C=5[H]										
**74**	4-(4-(*N*-((*S*)-1-(((2*S*,3*R*,4*S*)-1-cyclohexyl-3,4-dihydroxy-6-methylheptan-2-yl)amino)-1-oxo-3-phenylpropan-2-yl)sulfamoyl)piperazin-1-yl)-4-oxobutanoic acid	O=C(N([H])[C@@]([H])(C([H])([H])[C@@]1([H])C([H])([H])C([H])([H])C([H])([H])C([H])([H])C1([H])[H])[C@@]([H])(O[H])[C@@]([H])(O[H])C([H])([H])C([H])(C([H])([H])[H])C([H])([H])[H])[C@@]([H])(N([H])[S](=O)(=O)N2C([H])([H])C([H])([H])N(	-1.36	-0.40	0.97	-1.03	0.33	-1.23	0.14	-1.42	0.06	o	Stewart *et al.* 1995

C(=O)C([H])([H])C([H])([H])C(=O)O[H])C([H])([H])C2([H])[H])C([H])([H])C=3C([H])=C([H])C([H])=C([H])C=3[H]

Table S2. Optimal runtime parameters for the SVR models.

Parameter	SVR A	SVR B	SVR C	HSVR
SVM type	ε-SVR	ε-SVR	ε-SVR	ε-SVR
Kernal type	Radial basis function.	Radial basis function	Radial basis function	Radial basis function
γ	0.06	0.25	0.25	0.37
Cost	32.00	16.00	16.00	575.00
ε	0.25	0.25	0.06	0.16

Table S3. Confusion matrix for the qualitative predictive model.

		Observed	
		+	−
Predicted	+	true positive (TP)	false positive (FP)
	−	false negative (FN)	true negative (TN)

Table S4. The Cooper statistics and Kubat's G-mean calculated from the confusion matrix.

Parameter	Definition
Sensitivity (Se)	TP / (TP + FN)
Specificity (Sp)	TN / (FP +TN)
Accuracy (Acc)	(TP + TN) / (TP + TN + FP + FN)
Positive predictivity (PP)	TP / (TP + FP)
Negative predictivity (NP)	TN / (TN + FN)
Matthews Correlation Coefficient (MCC)	$\frac{\mathrm{TP}\times\mathrm{TN}-\mathrm{FP}\times\mathrm{FN}}{\sqrt{(\mathrm{TP}+\mathrm{FP})\times(\mathrm{TP}+\mathrm{FN})\times(\mathrm{TN}+\mathrm{FP})\times(\mathrm{TN}+\mathrm{FN})}}$
Geometric mean (*g*-mean)	(Sensitivity × Specificity)^{½}
Harmonic mean of sensitivity and positive predictivity (*F-measure*)	$F-measure=\frac{2}{PP^{-1}+Se^{-1}}$
Cohen's kappa	κ = (Accuracy – p_e) / (1 – p_e) $p_{\mathrm{True}}=\frac{\mathrm{TP}+\mathrm{FN}}{\mathrm{TP}+\mathrm{TN}+\mathrm{FP}+\mathrm{FN}}\cdot\frac{\mathrm{TP}+\mathrm{FP}}{\mathrm{TP}+\mathrm{TN}+\mathrm{FP}+\mathrm{FN}}$ $p_{\mathrm{False}}=\frac{\mathrm{TN}+\mathrm{FN}}{\mathrm{TP}+\mathrm{TN}+\mathrm{FP}+\mathrm{FN}}\cdot\frac{\mathrm{TN}+\mathrm{FP}}{\mathrm{TP}+\mathrm{TN}+\mathrm{FP}+\mathrm{FN}}$

$p_e = p_{True} + p_{False}$
